# Cytotoxicity against HL60 Cells of Ficifolidione Derivatives with Methyl, *n*-Pentyl, and *n*-Heptyl Groups

**DOI:** 10.3390/molecules24224081

**Published:** 2019-11-12

**Authors:** Hisashi Nishiwaki, Megumi Ikari, Satomi Fujiwara, Kosuke Nishi, Takuya Sugahara, Koichi Akiyama, Satoshi Yamauchi

**Affiliations:** 1 Graduate School of Agriculture, Ehime University, 3-5-7 Tarumi, Matsuyama, Ehime 790-8566, Japan; 2 ADRES Tarumi station, Ehime University, 3-5-7 Tarumi, Matsuyama, Ehime 790-8566, Japan

**Keywords:** phloroglucinol, ficifolidione, HL60, cytotoxicity

## Abstract

Ficifolidione, a natural insecticidal compound isolated from the essential oils of Myetaceae species, is a spiro phloroglucinol with an isobutyl group at the C-4 position. We found that ficifolidione showed cytotoxicity against cancer cells via apoptosis. Replacement of the isobutyl group by *n*-propyl group did not influence the potency, but the effect of the replacement of this group by a shorter or longer alkyl group on the biological activity remains unknown. In this study, ficifolidione derivatives with alkyl groups such as methyl, *n*-pentyl, and *n*-heptyl group—instead of the isobutyl group at the C-4 position—were synthesized to evaluate their cytotoxicity against the human promyelocytic leukaemia cell line HL60 and their insecticidal activity against mosquito larvae. The biological activities of their corresponding 4-epimers were also evaluated. As a result, the conversion of the isobutyl group to another alkyl group did not significantly influence the cytotoxicity or insecticidal activity. In HL60 cells treated with the *n*-heptyl-ficifolidione derivative, the activation of caspase 3/7 and the early stages of apoptosis were detected by using immunofluorescence and flow cytometric techniques, respectively, suggesting that the cytotoxicity should be induced by apoptosis even though the alkyl group was changed.

## 1. Introduction

Essential oils extracted from various kinds of plants have attracted great interest because the oils show various interesting biological activities, such as pest insect repellent, anti-fungal, antioxidant and cytotoxic activities [1,2,3,4]. Among these plants, the genus *Eucalyptus* are well-known as a source of various essential oils, including *p*-menthane-3,8-diol (repellent against mosquitoes from *E. citriodora*) [5] and ericifolione (insecticidal activity) [6]. Ficifolidione, tetramethyltetrahydrochromenedione-spirobicyclo[3.1.1]cylcoheptane, was reportedly isolated from two Myetaceae species, *Eucalyptus ficifolia* and *Kunzea ericoides*, using hexane extraction in the course of exploring insecticidal substances against worms and mosquitoes [7].

**Figure 1:**
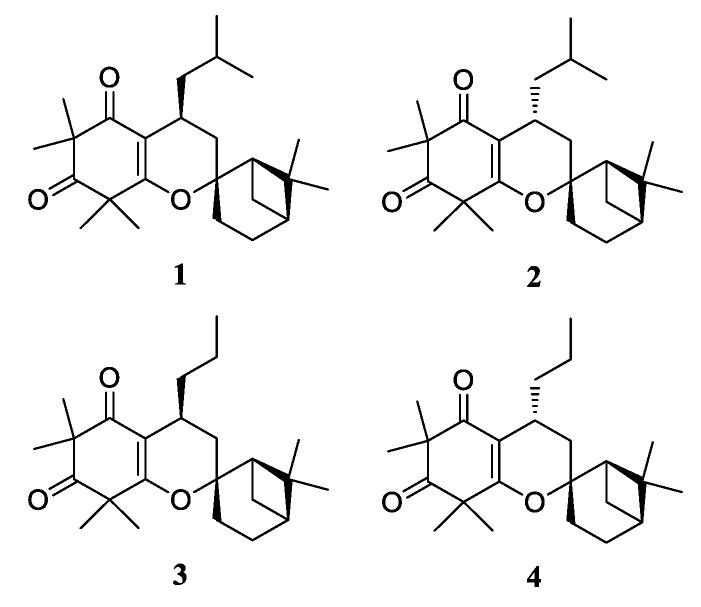
Structures of ficifolidione (**1**), its 4-epimer (**2**), and *n*-propyl derivatives (**3**) (4-*S* form), and (**4**) (4-*R* form) synthesized in our previous study [8].

We were interested in analysing the structure-activity relationship (SAR) of ficifolidione, a unique spiro-compound with a phloroglucinol moiety, using a series of derivatives of this compound. In the course of preparing ficifolidione, we revised the stereochemistry of ficifolidione not only by comparing the NMR and MS/MS data between natural and synthesized compounds but also by analysing the X-ray crystallographic structures of synthesized compounds [8]. Ficifolidione included in *kanuka*, the essential oil of *K. ericoides*, has a 4-*S* absolute configuration (**1** in Figure 1; its C-4 epimer **2** in Figure 1 had been previously reported as the natural configuration). After revising the stereochemistry, biological activity, such as insecticidal activity against houseflies, *Musca domestica*, mosquitoes, *Culex pipiens*, and cutworms, *Spodoptera litura,* and the cytotoxicity against four cell lines (insect cell (Sf9), mouse carcinoma cell (Colon26), human promyelocytic leukaemia cell (HL60), and African green monkey kidney epithelial cell (Vero) lines), were measured. We found that ficifolidione showed little insecticidal activity against the insects tested, whereas higher cell toxicity was observed against cancer cells compared with normal Vero cells. To analyse the cytotoxic mechanisms, DNA fragmentation and caspase 3 activation were investigated. Consequently, we observed both in the cancer cells treated with ficifolidione, demonstrating that the cell toxicity of ficifolidione should be induced via apoptosis. However, there was no evidence as to which phase of apoptosis was induced by ficifolidione.

On the other hand, we prepared an *n*-propyl derivative of ficifolidione and its C-4 epimer (**3** and **4** in Figure 1) to evaluate the effect of an alkyl group attached at the C-4 position on the cytotoxicity and found moderate cytotoxicity of these *n*-propyl-derivatives [8]. Since a modification of this moiety might influence the cell toxicity and strengthen the insecticidal activity against pest insects such as mosquitoes, SAR analyses of this moiety are necessary to elucidate the role of this alkyl group in exerting biological activity.

**Figure 2:**
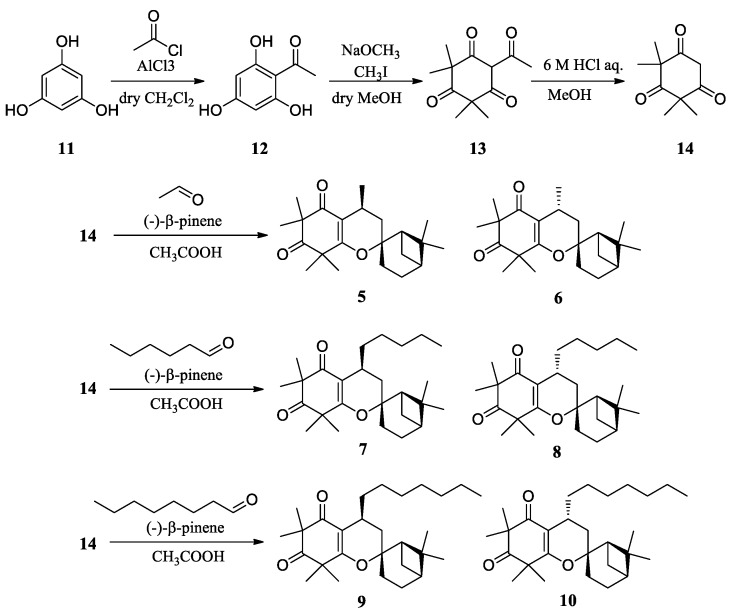
Synthetic scheme of ficifolidione derivatives containing an alkyl group instead of an isobutyl group at the C-4 position (*S*-Me (**5**), *R*-Me (**6**); *S*-*n*-pentyl (**7**), *R*-*n*-pentyl (**8**); *S*-*n*-heptyl (**9**), and *R*-*n*-heptyl (**10**)).

In this study, we prepared ficifolidione derivatives with shorter and longer alkyl groups instead of the isobutyl group and their corresponding C-4 epimers (**5**–**10** in Figure 2). Furthermore, the cytotoxic mechanism induced by the ficifolidione derivatives was investigated in detail by using immunofluorescence and flow cytometric analyses.

## 2. Results and Discussion

### 2.1. Synthesis of Ficifolidione Derivatives

Employing phloroglucinol **11** as the starting material, syncarpic acid **14** was prepared through the synthetic scheme shown in Figure 2. Ficifolidione derivatives were successfully synthesized using **14**, β-pinene and a corresponding aldehyde. Since both the target ficifolidione derivative and its C-4 epimer were synthesized at the same time through this synthetic scheme, these compounds were separated using a preparative HPLC system. After HPLC separation, their NMR and high-resolution MS spectra were analysed. We assigned the chemical configuration of the compounds according to the sign of their specific rotation values. The specific rotation values of compounds **5**, **7**, and **9** were +16, +9, and +17, respectively, to be assigned as the 4-*S* configuration, whereas those of compounds **6**, **8**, and **10** were −105, −84, and −87, respectively, to be assigned as the 4-*R* configuration. This is because the specific rotation values of 4-*S*-ficifolidione **1** and its propyl derivative **3** were +37 and +17, whereas those of their 4-epimers **2** and **4** were −92 and −26, respectively [8].

### 2.2. Insecticidal Activity against Mosquito Larvae

Ficifolidione was reportedly active against the larvae of *Aedes aegypti* and *C. quinquefasciatus* (48% and 30% mortality at 0.2 μg/insect) [7], whereas ficifolidione was inactive against *C. pipiens* larvae in our previous study [8]. The factors inducing the selectivity among these insects remain unknown. Ficifolidione derivatives with a different alkyl group might show larvicidal activity against *C. pipiens* because the permeability of the compounds through the membrane might be improved. We therefore examined the larvicidal activity of compounds **5**–**10** 24 h after application, but their apparent larvicidal activity against mosquitoes was not observed at a concentration of 50 μM, as shown in Table 1. We did not prepare a higher concentration than 50 μM because of the low solubility. The log P values, a hydrophobic parameter, of the methyl-, pentyl- and heptyl ficifolidione derivatives were 3.69, 4.85, and 6.19, respectively. (-)-Dihydroguaiaretic acid (log P, 5.01), a natural lignan compound, can kill over 50% of the *C*. *pipiens* larvae at the same concentration (LC_50_ value is 35 μM) [9]. Taking this into consideration, the inactiveness of the compounds against *C*. *pipiens* suggests that the alkyl moiety and core structure of ficifolidione should not interact with any target sites in *C*. *pipiens*.

### 2.3. Cytotoxicity against HL60 Cells

The cytotoxicity against the tumour cell line HL60 was evaluated (Table 2). Most of the ficifolidione derivatives showed high cytotoxicity against the tumour cell lines (IC_50_ values below 10 μM). The IC_50_ values of the *n*-propyl derivatives were slightly larger, but the potency did not depend on the length of the alkyl group. Comparing the IC_50_ values between the 4-*S* and 4-*R* configurations, the 4-*S* forms were likely to show higher activity, although the differences in the potencies were statistically insignificant. These results suggest that the length of the alkyl group and stereostructure at the C-4 position should not influence cytotoxicity.

### 2.4. Immunofluorescence Analysis

**Figure 3:**
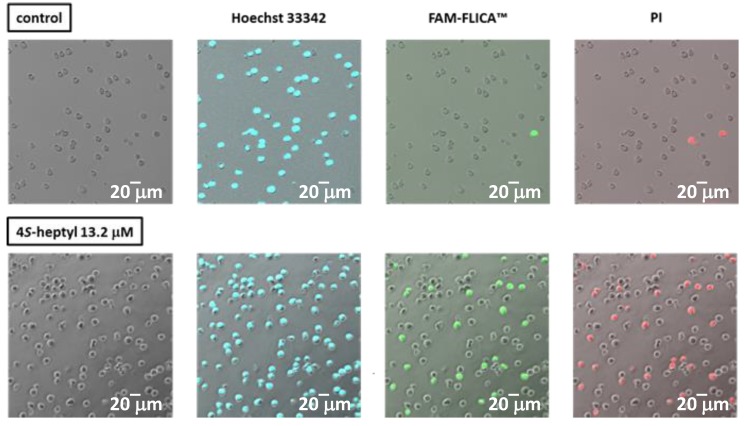
Fluorescence staining of HL60 cells treated with 4*S*-*n*-heptyl derivative **9**.

We already reported that DNA fragmentation and caspase 3 activation were induced by ficifolidione in cancer cells [8]. In the present research, we examined whether the modification of the alkyl group influenced apoptosis induction by employing immunofluorescence analysis (Figure 3). The 4-*S*-*n*-heptyl derivative **9** was selected as the test compound because the larger flexibility of the *n*-heptyl group compared with the other alkyl groups might influence the apoptotic potency, and the *S* configuration of this compound is the same as that of natural ficifolidione. Staining by Hoechst 33342 revealed a blue chromatin condensation pattern in the live and dead HL60 cell nuclei. After treatment with the 4-*S*-*n*-heptyl derivative **9** (13.2 μM; twice the IC_50_ value), the application of propidium iodide (PI) stained some cells as red, indicating dead cells (26% of the cells were stained). On the other hand, activated caspase 3/7 was observed as a green staining in the cells treated with the heptyl ficifolidione derivative (21% of the cells were stained). Since we observed these cells 24 h after application, the percentage of the stained cells was 20–30%, but most of the red-stained cells were also stained green as shown in Figure 3, demonstrating that compound **9** as well as ficifolidione induced activated caspase 3/7, resulting in apoptosis.

### 2.5. Flow Cytometric Analysis

**Figure 4:**
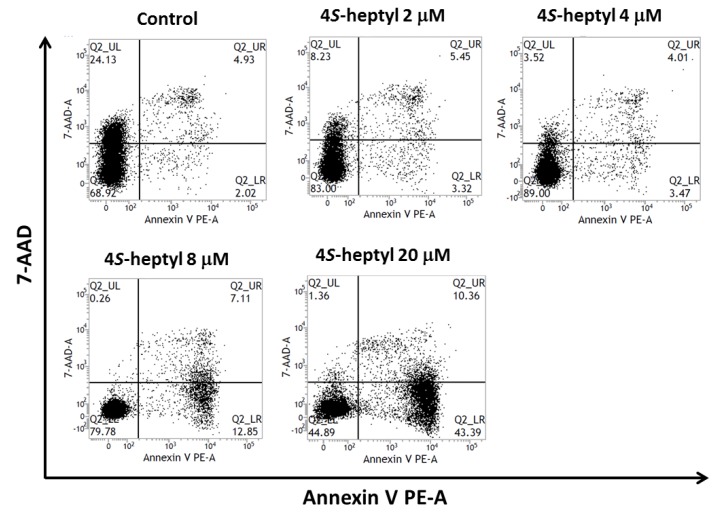
Flow cytometry using HL60 cells treated with 4*S*-*n*-heptyl derivative **9**. Cells were stained with annexin V and 7-AAD before flow cytometry analysis. The percentage of early and late stage apoptotic cells are indicated in Q2_LR and Q2_UR, respectively.

The mechanism of cell death induction was investigated by using flow cytometry (Figure 4). In this experiment, HL60 cells were incubated with 4-S-*n*-heptyl derivative **9**. The percentage of apoptotic cells was determined by double staining with annexin V/7-AAD. The ratio of early apoptotic cells treated with compound **9** was higher than that of the control in a dose-dependent manner (3.32% at 2 μM to 43.39% at 20 μM), suggesting that the *n*-heptyl derivative **9** induced HL60 cells to early-stage apoptosis.

## 3. Materials and Methods

### 3.1. Chemicals

Reagents used for the syntheses and evaluation of the various biological activities were purchased from Fujifilm Wako Pure Chemical Co. (Osaka, Japan), Nacalai Tesque, Inc. (Kyoto, Japan), Tokyo Chemical Industry Co, Ltd. (Tokyo, Japan), and Sigma-Aldrich Japan K. K. (Tokyo, Japan). Melting points were uncorrected. Optical rotations were measured on a SEPA-200 instrument (Horiba Ltd., Kyoto, Japan). NMR data were obtained using an ECS400 spectrometer (JEOL Resonance Inc., Tokyo, Japan). HR-MS data were obtained using Xevo-Q-TOF MS. The instrument was operated with an ESI source in positive ion mode. Mass determination was performed using the following MS conditions: Capillary voltage, 3.0 kV; desolvation temperature, 400 °C; and desolvation gas flow rate, 1000 L/h. High purity argon (99.99%) was used as the collision gas at a flow rate of 1.5 mL/min. Mass spectra were collected in the range of *m*/*z* 100 to 1000. Log P values were calculated using ChemBioDraw Ultra software version 12 (PerkinElmer Japan Co., Ltd. (Yokohama, Japan)).

### 3.2. Synthesis of Ficifolidione Derivatives

Ficifolidione derivatives (**5**–**10**, Figure 2) were synthesized via a similar procedure as reported previously [8]. In brief, phloroglucinol (**11**) was employed as the starting material, and syncarpic acid (**14**) was prepared via intermediates **12** and **13**. In addition, syncarpic acid, (-)-β-pinene and a variety of aldehydes, such as acetaldehyde, hexanal or octanal, were condensed and cyclized using a Diels-Alder reaction. From the resulting compounds, the 4*S*- and 4*R*-derivatives were separately collected using silica gel column chromatography and HPLC. H-NMR, C-NMR, DEPT, DEPT (expanded), HMQC and HMBC spectra of compounds **5**–**10** were available online as Supplementary Materials (Figures S1–S36).

#### 3.2.1. 4-Methyl ficifolidione 4S **5** and 4R **6**

A reaction mixture containing syncarpic acid (**14**, 0.40 g, 2.2 mmol), potassium acetate (0.02 g, 0.20 mmol), acetic acid (10 mL), 4A molecular sieves (3 g), acetaldehyde (0.19 g, 4.39 mmol), and (-)-β-pinene (1.16 g, 8.78 mmol) was stirred for 22 h at room temperature. The solvent was removed in vacuo, and the residue was suspended in 20 mL CH_2_Cl_2_, and filtered. The filtrate was washed with H_2_O. The organic layer was dried over anhydrous Na_2_SO_4_, and the solvent was removed in vacuo. The residue was separated by silica gel column chromatography (*n*-hexane:Et_2_O, 10:1). Preparative HPLC (COSMOSIL 5C18-MS-II (20 mm i.d. × 250 mm) with a guard column (10 mm i.d. × 20 mm) and mobile phase of H_2_O:MeOH = 10:90 with a flow rate of 2.0 mL/min) afforded **5** (0.04 g, 5%) and **6** (0.02 g, 5%).

**5**: White crystals; mp 77–78 °C; [α]^25^_D_ + 16 (c 1.0 CHCl_3_); ^1^H NMR (CDCl_3_, 400 MHz) δ 2.67 (1H, m, H-4), 2.21 (1H, d, *J* = 6 Hz, H-3b), 2.14–2.18 (2H, m, H-1′ and H-7′b), 2.09–2.13 (1H, m, H-3′b), 1.99–2.06 (1H, m, H-5′), 1.95–1.98 (1H, m, H-4′b), 1.89–1.94 (1H, m, H-3′a), 1.87–1.89 (1H, m, H-4′a), 1.60 (1H, dd, *J* = 11, 14 Hz, H-3a), 1.50 (1H, d, *J* = 9.2 Hz, H-7′a), 1.40 (3H, s, H-9 or H-10), 1.32 (3H, s, H-11 or H-12), 1.29 (3H, s, H-11 or H-12), 1.27 (3H, s, H-9 or H-10), 1.24 (3H, s, H-9′), 1.13 (3H, d, *J* = 6.4 Hz, H-1′′), 0.98 (3H, s, H-8′); ^13^C NMR (CDCl_3_, 100 MHz) δ 213.6 (C, C-7), 198.1 (C, C-5), 169.4 (C, C-8a), 112.5 (C, C-4a), 84.8 (C, C-2), 55.3 (C, C-6), 48.0 (CH, C-8), 46.5 (C, C-1′), 42.5 (CH_2_, C-3), 40.5 (CH, C-5′), 38.2 (C, C-6′), 30.3 (CH_2_, C-3′), 27.5 (CH_3_, C-9′), 26.2 (CH_3_, C-9 or C-10), 26.07 (CH_3_, C-11 or C-12), 26.07 (CH_2_, C-7′), 24.7 (CH_2_, C-4′), 24.0 (CH_3_, C-9 or C-10), 23.7 (CH, C-4), 23.1 (CH_3_, C-8′), 22.6 (CH_3_, C-11 or C-12), 19.3 (CH_3_, C-1′′), HR-ESI-MS *m*/*z* 345.2469 (calculated for C_2__2_H_3__3_O_3_, 345.2430).

**6**: White crystals; mp 66–68 °C; [α]^25^_D_ − 105 (c 1.0 CHCl_3_); ^1^H NMR (CDCl_3_, 400 MHz) δ 2.77 (1H, m, H-4), 2.25–2.30 (1H, m, H-7′b), 2.05–2.10 (2H, m, H-3b and H-1′), 1.93–2.00 (1H, m, H-5′), 1.85–1.94 (2H, m, H-3′), 1.73–1.82 (2H, m, H-4′), 1.60 (1H, d, *J* = 10.0 Hz, H-7′a), 1.41 (1H, dd, *J* = 11, 14 Hz, H-3a), 1.36 (3H, s, H-11 or H-12), 1.33 (3H, s, H-9 or H-10), 1.33 (3H, s, H-9 or H-10), 1.31 (3H, s, H-11 or H-12), 1.30 (3H, s, H-9′), 1.15 (3H, d, *J* = 6.8 Hz, H-1′′), 1.01 (3H, s, H-8′); ^13^C NMR (CDCl_3_, 100 MHz) δ 213.6 (C, C-7), 198.0 (C, C-5), 169.7 (C, C-8a), 113.2 (C, C-4a), 84.3 (C, C-2), 55.5 (C, C-6), 51.7 (C, C-1′), 48.0 (CH, C-8), 43.0 (CH_2_, C-3), 40.4 (CH, C-5′), 38.2 (C, C-6′), 27.6 (CH_2_, C-4′), 27.4 (CH_3_, C-9′), 26.9 (CH_2_, C-7′), 26.3 (CH_3_, C-11 or C-12), 25.5 (CH_3_, C-9 or C-10), 24.9 (CH_2_, C-3′), 24.3 (CH_3_, C-11 or C-12), 23.5 (CH_3_, C-8′), 22.7 (CH, C-4), 22.3 (CH_3_, C-9 or C-10), 19.5 (CH_3_, C-1′′), HR-ESI-MS *m*/*z* 345.2453 (calculated for C_2__2_H_3__3_O_3_, 345.2430).

#### 3.2.2. 4-Pentyl Ficifolidione 4S **7** and 4R **8**

**7** (0.11 g, 9%): Oil, [α]^25^_D_ + 9 (c 1.0 CHCl_3_); ^1^H NMR (CDCl_3_, 400 MHz) δ 2.59 (1H, dd, *J* = 16, 7 Hz, H-4), 2.22 (1H, dd, *J* = 14, 7 Hz, H-3b), 2.10–2.18 (3H, m, H-1′, H-3′ or H-4′ and H-7′b), 2.00–2.10 (2H, m, H-3′ and H-4′), 1.94–1.97 (1H, m, H-5′), 1.83–1.92 (2H, m, H-3′ or H-4′, and H-1′′b), 1.64 (1H, dd, *J* = 11, 14 Hz, H-3a), 1.49 (1H, d, *J* = 9.2 Hz, H-7a′), 1.32–1.41 (3H, m, H-3′′ or H-4′′, and H-1′′a), 1.39 (3H, s, H-9 or H-10), 1.32 (3H, s, H-11 or H-12), 1.29 (3H, s, H-11 or H-12), 1.29 (2H, m, H-2′′), 1.26 (2H, m, H-3′′ or H-4′′), 1.26 (3H, s, H-9 or 10), 1.24 (3H, s, H-9′), 0.99 (3H, s, H-8′), 0.88 (3H, t, *J* = 7 Hz, H-5′′); ^13^C NMR (CDCl_3_, 100 MHz) δ 213.2 (C, C-7), 198.1 (C, C-5), 169.8 (C, C-8a), 111.8 (C, C-4a), 84.9 (C, C-2), 55.2 (C, C-6), 48.1 (C, C-8), 46.5 (CH, C-1′), 40.5 (CH, C-5′), 39.0 (CH_2_, C-3), 38.1 (C, C-6′), 32.3 (CH_2_, C-1′′), 31.8 (CH_2_, C-3′′ or C-4′′), 30.3 (CH_2_, C-3′ or C-4′), 28.3 (CH, C-4), 27.5 (CH_3_, C-9′), 26.14 (CH_3_, C-9 or C-10), 26.05 (CH_2_ x 2, C-2′′ and C-7′), 25.9 (CH_3_, C-11 or C-12), 24.6 (CH_2_, C-3′ or C-4′), 24.0 (CH_3_, C-9 or C-10), 23.1 (CH_3_, C-8′), 22.7 (CH_3_, C-11 or C-12), 22.6 (CH_2_, C-3′′ or C-4′′), 14.0 (CH_3_, C-5′′); HR-ESI-MS *m*/*z* 401.3030 (calculated for C_2__6_H_41_O_3_, 401.3056).

**8** (0.10 g, 9%): Oil, [α]^25^_D_ − 84 (c 1.0 CHCl_3_); ^1^H NMR (CDCl_3_, 400 MHz) δ 2.67 (1H, dd, *J* = 16, 9 Hz, H-4), 2.25–2.30 (1H, m, H-7′b), 2.10 (1H, dd, *J* = 5 Hz, H-3b), 2.07 (1H, m, H-1′), 1.96–2.00 (1H, m, H-5′), 1.80–1.93 (4H, m, H-3′ and H-4′), 1.69–1.85 (1H, m, H-1′′b), 1.61 (1H, d, *J* = 10 Hz, H-7a′), 1.48 (1H, dd, *J* = 10, 14 Hz, H-3a), 1.33–1.37 (1H, m, H-1′′a), 1.36 (3H, s, H-11 or H-12), 1.33 (6H, s, H-9 and H-10), 1.30 (3H, s, H-11 or H-12), 1.29 (3H, s, H-9′), 1.20–1.27 (6H, m, H-2′′, H-3′′ and H-4′′), 1.01 (3H, s, H-8′), 0.88 (3H, t, *J* = 7 Hz, H-5′′); ^13^C NMR (CDCl_3_, 100 MHz) δ 213.7 (C, C-7), 198.0 (C, C-5), 169.1 (C, C-8a), 112.6 (C, C-4a), 84.3 (C, C-2), 55.4 (C, C-6), 51.6 (CH, C-1′), 48.0 (C, C-8), 40.4 (CH, C-5′), 39.5 (CH_2_, C-3), 38.2 (C, C-6′), 32.5 (CH_2_, C-1′′), 31.9 (CH_2_, C-3′′), 27.7 (CH_2_, C-3′ or C-4′), 27.5 (CH, C-4), 27.4 (CH_3_, C-9′), 26.9 (CH_2_, C-7′), 26.3 (CH_3_, C-11 or C-12), 26.2 (CH_2_, C-2′′), 25.5 (CH_3_, C-9 or C-10), 24.9 (CH_2_, C-3′ or C-4′), 24.4 (CH_3_, C-11 or C-12), 23.4 (CH_3_, C-8′), 22.7 (CH_2_, C-4′′), 22.4 (CH_3_, C-9 or C-10), 14.1 (CH_3_, C-5′′); HR-ESI-MS *m*/*z* 401.3097 (calculated for C_2__6_H_41_O_3_, 401.3056).

#### 3.2.3. 4-Heptyll ficifolidione 4S **9** and 4R **10**

**9** (0.19 g, 14%): White crystals; mp 47–49 °C; [α]^25^_D_ + 17 (c 1.0 CHCl_3_); ^1^H NMR (CDCl_3_, 400 MHz) δ 2.52 (1H, dd, *J* = 17, 10 Hz, H-4), 2.14 (1H, dd, *J* = 14, 6 Hz, H-3b), 2.00–2.10 (3H, m, H-3′b, H-7′b and H-1′), 1.92–1.93 (1H, m, H-4′b), 1.81–1.92 (2H, m, H-3′a and H-5′), 1.76–1.78 (3H, m, H-1′′ and H-4′a), 1.57 (1H, dd, *J* = 14, 11 Hz, H-3a), 1.42 (1H, d, *J* = 9 Hz, H-7a′), 1.32 (3H, s, H-9 or H-10), 1.11–1.28 (10H, m, H-2′′, H-3′′, H-4′′, H-5′′ and H-6′′), 1.24 (3H, s, H-11 or H-12), 1.21 (3H, s, H-11 or H-12), 1.19 (3H, s, H-9 or H-10), 1.17 (3H, s, H-9′), 0.91 (3H, s, H-8′), 0.80 (3H, t, *J* = 6 Hz, H-7′′); ^13^C NMR (CDCl_3_, 100 MHz) δ 213.5 (C, C-7), 198.0 (C, C-5), 169.8 (C, C-8a), 111.8 (C, C-4a), 84.8 (C, C-2), 55.2 (C, C-6), 48.2 (C, C-8), 46.5 (CH, C-1′), 40.5 (CH, C-5′), 39.1 (CH_2_, C-3), 38.2 (C, C-6′), 32.4 (CH_2_, C-1′′), 31.8 (CH_2_, C-5′′ or C-6′′), 30.3 (CH_2_, C-3′), 29.6 (CH_2_, C-2′′, C-3′′ or C-4′′), 29.3 (CH_2_, C-2′′, C-3′′ or C-4′′), 28.3 (CH, C-4), 27.4 (CH_3_, C-9′), 26.4 (CH_2_, C-2′′, C-3′′ or C-4′′), 26.1 (CH_3_, C-9 or C-10), 26.0 (CH_2_, C-7′), 25.9 (CH_3_, C-11 or C-12), 24.6 (CH_2_, C-4′), 24.0 (CH_3_, C-9 or C-10), 23.1 (CH_3_, C-8′), 22.7 (CH_3_, C-11 or C-12), 22.5 (CH_2_, C-5′′ or C-6′′), 14.0 (CH_3_, C-7′′); HR-ESI-MS *m*/*z* 429.3394 (calculated for C_2__8_H_45_O_3_, 429.3369).

**10** (0.10 g, 8%): Oil, [α]^25^_D_ − 87 (c 1.0 CHCl_3_); ^1^H NMR (CDCl_3_, 400 MHz) δ 2.66 (1H, dd, *J* = 16, 7 Hz, H-4), 2.24–2.30 (1H, m, H-7′b), 2.10 (1H, t, *J* = 5 Hz, H-1′), 2.06 (1H, d, *J* = 6 Hz, H-3b), 1.98–2.00 (1H, m, H-5′), 1.85–1.93 (2H, m, H-4′), 1.74–1.85 (3H, m, H-1′′b and H-3′), 1.61 (1H, d, *J* = 10 Hz, H-7a′), 1.47 (1H, dd, *J* = 14, 10 Hz, H-3a), 1.37–1.25 (10H, m, H-2′′, H-3′′, H-4′′, H-5′′ and H-6′′), 1.36 (3H, s, H-9 or H-10), 1.33 (3H, s, H-9 or H-10), 1.32 (3H, s, H-11 or H-12), 1.30 (3H, s, H-11 or H-12), 1.29 (3H, s, H-9′), 1.19–1.29 (1H, m, H-1′′a), 1.01 (3H, s, H-8′), 0.87 (3H, t, *J* = 6 Hz, H-7′′); ^13^C NMR (CDCl_3_, 100 MHz) δ 213.7 (C, C-7), 198.0 (C, C-5), 169.1 (C, C-8a), 112.6 (C, C-4a), 84.3 (C, C-2), 55.5 (C, C-6), 51.6 (CH, C-1′), 48.1 (C, C-8), 40.5 (CH, C-5′), 39.7 (CH_2_, C-3), 38.3 (C, C-6′), 32.6 (CH_2_, C-1′′), 31.9 (CH_2_, C-5′′ or C-6′′), 29.7 (CH_2_, C-2′′, C-3′′ or C-4′′), 29.4 (CH_2_, C-2′′, C-3′′ or C-4′′), 27.7 (CH_2_, C-3′), 27.6 (CH, C-4), 27.4 (CH_3_, C-9′), 26.9 (CH_2_, C-7′), 26.6 (CH_2_, C-2′′, C-3′′ or C-4′′), 26.3 (CH_3_, C-11 or C-12), 25.5 (CH_3_, C-9 or C-10), 24.9 (CH_2_, C-4′), 24.4 (CH_3_, C-9 or C-10), 23.4 (CH_3_, C-8′), 22.6 (CH_2_, C-5′′ or C-6′′), 22.4 (CH_3_, C-11 or C-12), 14.1 (CH_3_, C-7′′); HR-ESI-MS *m*/*z* 429.3387 (calculated for C_2__8_H_45_O_3_, 429.3369).

### 3.3. Evaluation of Insecticidal Activity against Mosquito Larvae

The assay method against mosquito larvae was essentially the same as in our previous report [9]. Eggs of *C. pipiens* were purchased from Sumika Technoservice Co. (Takarazuka, Japan) and reared. To evaluate the larvicidal activity against mosquitoes, 5 μL of a DMSO solution containing ficifolidione was added to 1 mL of water (the final concentration was adjusted to 50 μM), into which 10 of the third instar larvae were released. Twenty larvae (10 × 2 tubes) were used for each insecticidal assay, and insecticidal activity was monitored within 24 h after application.

### 3.4. Cell Proliferation and Viability Assays

Anti-proliferative activity was evaluated by a water-soluble tetrazolium salt-8 (WST-8) assay (Kishida Chemical, Osaka, Japan) according to the manufacturer′s instructions. The assay method was essentially the same as in our previous report [8]. In brief, the number of HL60 cells in Roswell Park Memorial Institute (RPMI) medium supplemented with 2% FBS was adjusted to 1 × 10^4^ cells/well in a 96-well culture plate (BD Falcon, Franklin Lakes, NJ, USA) and treated with various concentrations of test samples (final concentrations, 100–0.01 μM). After cultivating for 48 h in a CO_2_ incubator, the WST-8 solution was added to the culture media at a final concentration of 10% (*v*/*v*), and the cells were further cultured for 4 h. Then, the absorbance was monitored at 450 nm using a microplate reader (Model 680, Bio-Rad). The IC_50_ values (M), the concentration for inducing death in 50% of the sample-treated cells were calculated from the sigmoid curve using Prism version 5.0 (GraphPad Software, San Diego, CA, USA). The IC_50_ values are presented as the mean values obtained from at least three separate assays performed in triplicate.

### 3.5. Immunofluorescence Analysis

Immunofluorescence analysis was performed twice by using a FAM-FLICA Caspase-3/7 Assay Kit (Immunochemistry Technologies LLC (Bloomington, MN, USA)) according to the manufacturer′s instructions and our previous report [10]. HL60 cells in 2% FBS-RPMI in 5 mL dishes (1 × 10^6^ cells/dish) were treated with 0.5% DMSO (negative control) and compound **9** (final concentration, 13.2 μM). After incubation for 24 h in a CO_2_ incubator, the cells were harvested and resuspended in 10% FBS-RPMI, followed by the addition of FAM-FLICA. After incubation at 37 °C for 1 h protected from light, cells were exposed to Hoechst 33342 at 37 °C for 5 min and then washed with apoptosis wash buffer (Immunochemistry Technologies LLC). The suspension of cells in apoptosis wash buffer was subsequently incubated with PI for 5 min. After washing, the cells were analysed under a confocal microscope (Fluoview FV10i, Olympus (Tokyo, Japan)).

### 3.6. Flow Cytometric Analysis

Flow cytometric analysis was performed using an Annexin V-PE Apoptosis Detecting Kit I (BD Pharmingen, San Diego, CA, USA) according to the manufacturer′s instructions and our previous reports [11,12]. HL60 cells in 2% FBS-RPMI in 5 mL dishes (1 × 10^6^ cells/dish) were treated with 0.5% DMSO (negative control) or compound **9** (final concentration, 2–20 μM). After incubation for 48 h in a CO_2_ incubator, the harvested cells were resuspended in PBS (pH 7.4) to adjust the number of cells to 1 × 10^6^ cells/mL. PE Annexin V and 7-amino-actinomycin D were added, and the mixtures were incubated for 15 min protected from light. After adding the Annexin V binding buffer and 10× concentrate (BD Pharmingen, BD Biosciences (San Jose, CA, USA)), the cells were subjected to a BD FACSVerse flow cytometer (BD Biosciences (San Jose, CA, USA)).

## 4. Conclusions

The ficifolidione derivatives prepared in this study did not show any larvicidal activity against *C. pipiens*, whereas they did show high cytotoxicity. In addition, their cytotoxicity was found to be induced via early apoptosis. The IC_50_ values of *n*-propyl derivatives were slightly bigger, but the potency did not depend on the length of the alkyl group. The exchange to longer alkyl groups such as pentyl and heptyl groups did not influence cytotoxicity, suggesting that this moiety and the stereochemistry at this position should not be recognized by any receptor/enzyme proteins in HL60 cells and might be oriented to the outside of the protein surface. It should be suggested that the cytotoxicity depends on the core structure of ficifolidione. Recently, natural compounds having a phenyl group instead of the isobutyl group of ficifolidione were isolated from the Australian tree, *Corymbia intermedia* [13]. The cytotoxicity against human embryonic kidney cells (HEK293) of ficifolidione and its phenyl derivatives was evaluated as inactive at 40 and 100 μM, respectively [13,14]. The antiplasmodial activity of these compounds against the malaria parasite, *Plasmodium falciparum,* was also evaluated, and it was suggested that the replacing the isobutyl with phenyl group has no effect on the activity. The target proteins are still unknown, but this alkyl moiety could be used to introduce any spacer arm to catch a target protein by using affinity column chromatography which is an ongoing project. Further research could be useful for the identification of a target protein of cytotoxic ficifolidione. The successful development of novel pharmaceuticals could follow.

## Figures and Tables

**Table 1:** Mortality of mosquito larvae after application of ficifolidione derivatives (mean ± SEM, *n* = 4).

	5 4*S*-Me	6 4*R*-Me	7 4*S*-Pentyl	8 4*R*-Pentyl	9 4*S*-Heptyl	10 4*R*-Heptyl
Mortality (%)	6.2 ± 3.8	0 ± 0	0 ± 0	0 ± 0	3.8 ± 2.4	6.3 ± 2.4

**Table 2:** Cytotoxicity against the HL60 cell of ficifolidione and its derivatives (IC_50_ (μM); mean ± SEM).

	Me	*n*-Pro [8]	Isobutyl [8]	*n*-Pentyl	*n*-Heptyl
*S*	3.3 ± 0.2 (*n* = 3) ^a^	19.4 ± 2.1 (*n* = 5) ^a,b^	2.9 ± 0.3 (*n* = 3) ^a^	3.2 ± 0.7 (*n* = 3) ^a^	6.6 ± 0.03 (*n* = 7) ^a^
*R*	13.9 ± 2.2 (*n* = 3) ^a^^,^^b^	23.5 ± 8.3 (*n* = 5) ^b^	3.7 ± 0.9 (*n* = 3) ^a^	4.9 ± 1.0 (*n* = 3) ^a^	8.5 ± 0.4 (*n* = 7) ^a^

The different letters mean the significant difference (*p* < 0.05, one-way ANOVA, post-hoc test Tukey).

## Supplementary Materials

The following are available online at https://www.mdpi.com/1420-3049/24/22/4081/s1, Figures S1: H-NMR, C-NMR, DEPT, DEPT (expanded), HMQC and HMBC spectra of compounds **5**–**10**.
