# Supplementary material for: Cytotoxicity against HL60 Cells of Ficifolidione Derivatives with Methyl, n-Pentyl, and n-Heptyl Groups
Source: Molecules. 2019 Nov 12;24(22):4081. doi: 10.3390/molecules24224081 (PMC6891661; doi:10.3390/molecules24224081)

Supplementary Materials

Cytotoxicity against HL60 Cells of Ficifolidione Derivatives with Methyl, *n*-Pentyl, and *n*-Heptyl Groups

Hisashi Nishiwaki [1,*], Megumi Ikari [1], Satomi Fujiwara [1], Kosuke Nishi [1], Takuya Sugahara [1], Koichi Akiyama [2] and Satoshi Yamauchi [1]
[1]Graduate School of Agriculture, Ehime University, 3-5-7 Tarumi, Matsuyama, 790-8566 Ehime, Japan;
[2]ADRES Tarumi station, Ehime University, 3-5-7 Tarumi, Matsuyama, 790-8566 Ehime, Japan
*****Correspondence: nishiwaki.hisashi.mg@ehime-u.ac.jp; Tel.:+81-89-946-9973

Figures S1-S6 *S*-4-Methyl-ficifolidione (**5**)
H-NMR (S1), C-NMR (S2), DEPT (S3), DEPT (expanded) (S4), HMQC (S5), HMBC (S6)

Figures S7-S12 *R*-4-Methyl-ficifolidione (**6**)
H-NMR (S7), C-NMR (S8), DEPT (S9), DEPT (expanded) (S10), HMQC (S11), HMBC (S12)

Figures S13-18 *S*-4-Pentyl-ficifolidione (**7**)
H-NMR (S13), C-NMR (S14), DEPT (S15), DEPT (expanded) (S16), HMQC (S17), HMBC (S18)

Figures S19-S24 *R*-4-Pentyl-ficifolidione (**8**)
H-NMR (S19), C-NMR (S20), DEPT (S21), DEPT (expanded) (S22), HMQC (S23), HMBC (S24)

Figures S25-30 *S*-4-Heptyl-ficifolidione (**9**)
H-NMR (S25), C-NMR (S26), DEPT (S27), DEPT (expanded) (S28), HMQC (S29), HMBC (S30)

Figures S31-S36 *R*-4-Heptyl-ficifolidione (**10**)
H-NMR (S31), C-NMR (S32), DEPT (S33), DEPT (expanded) (S34), HMQC (S35), HMBC (S36)

Figure S1

S-4-Me-Ficifolidione (**5**) H-NMR

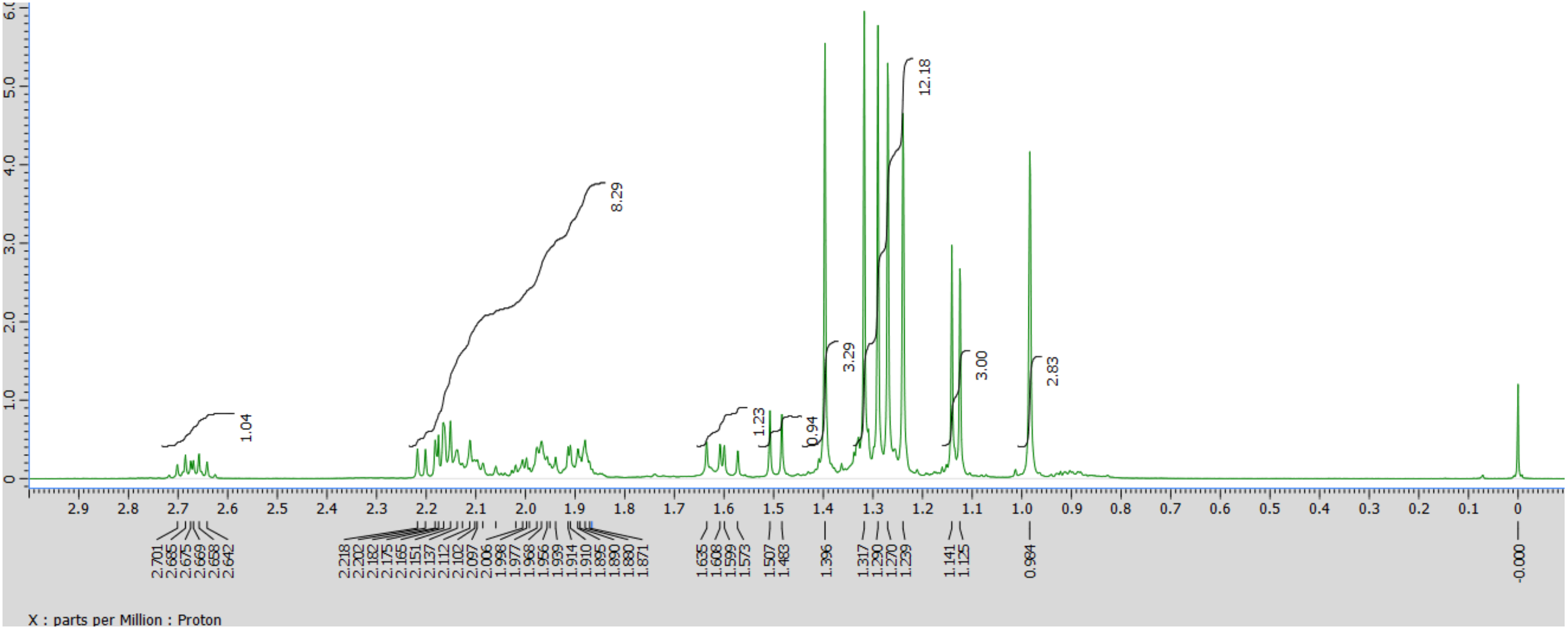

Figure S2

S-4-Me-Ficifolidione (**5**) C-NMR

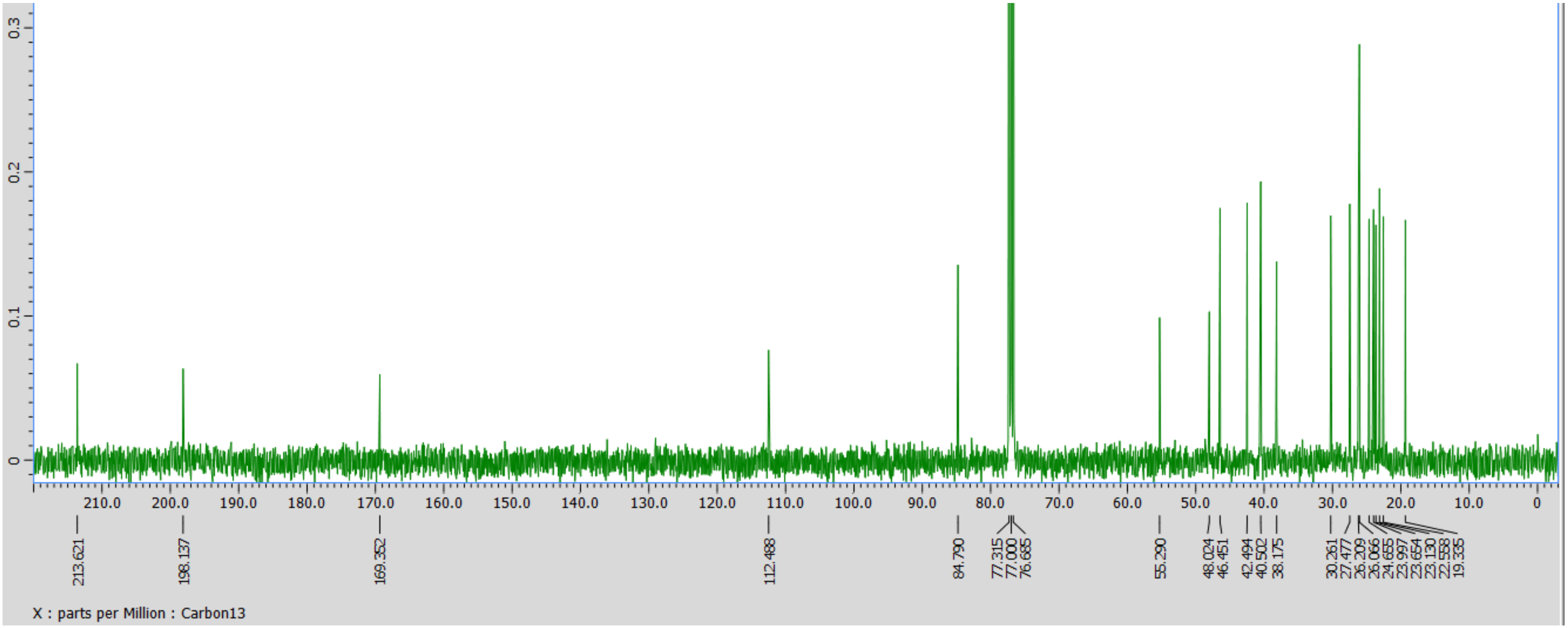

Figure S3

S-4-Me-Ficifolidione (**5**) DEPT

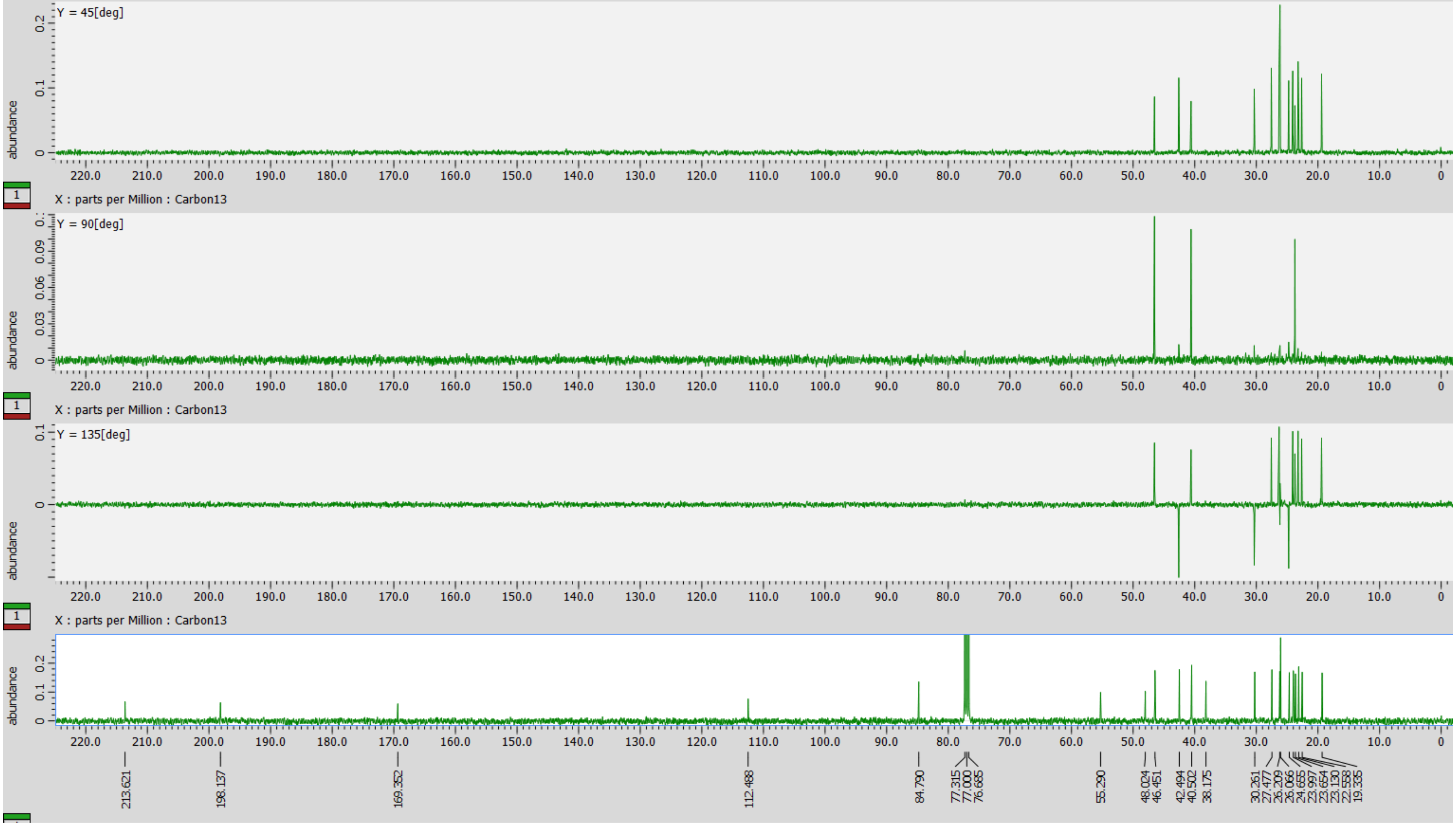

Figure S4

S-4-Me-Ficifolidione (**5**) DEPT expanded

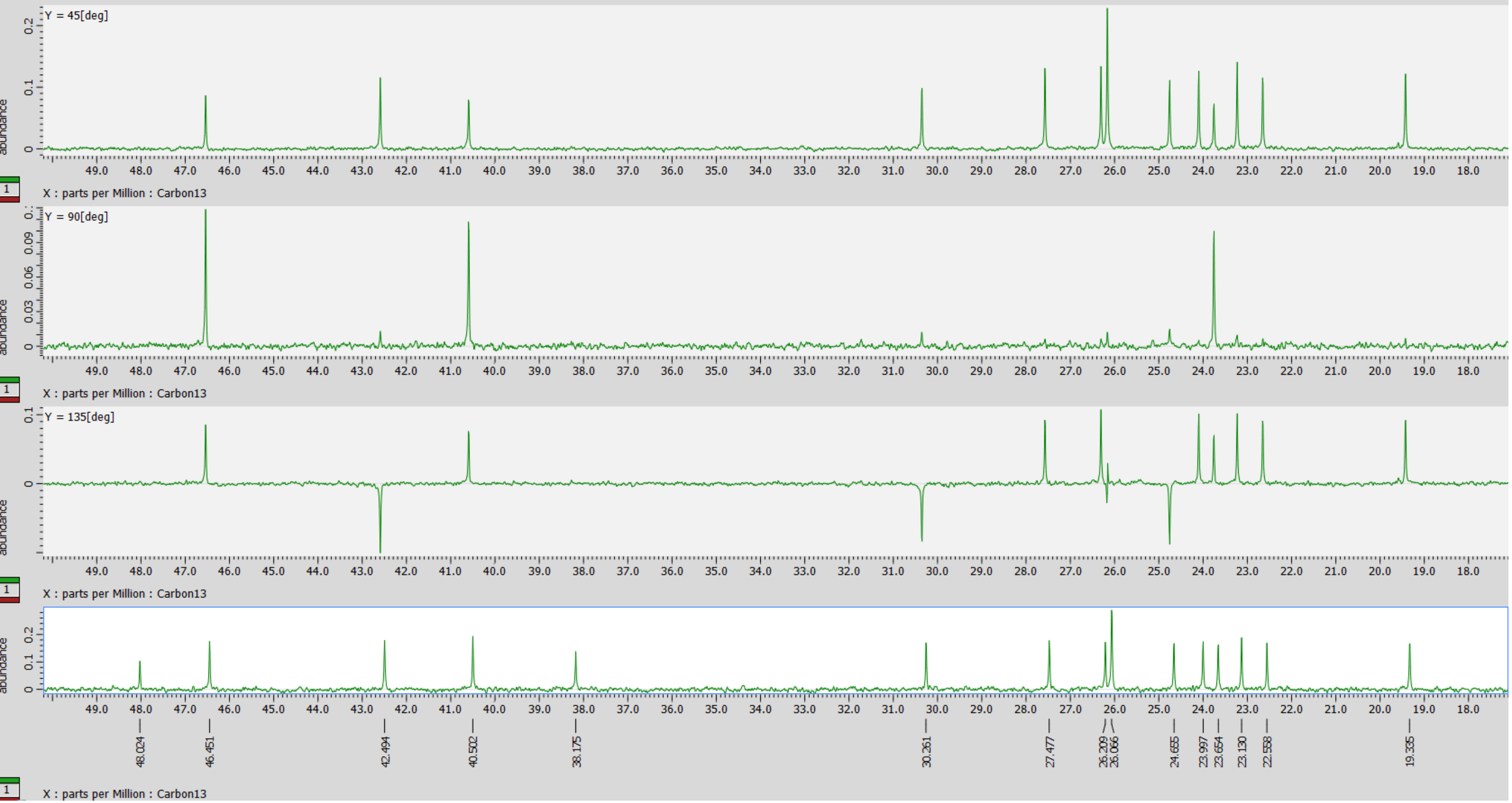

Figure S5

S-4-Me-Ficifolidione (**5**) HMQC

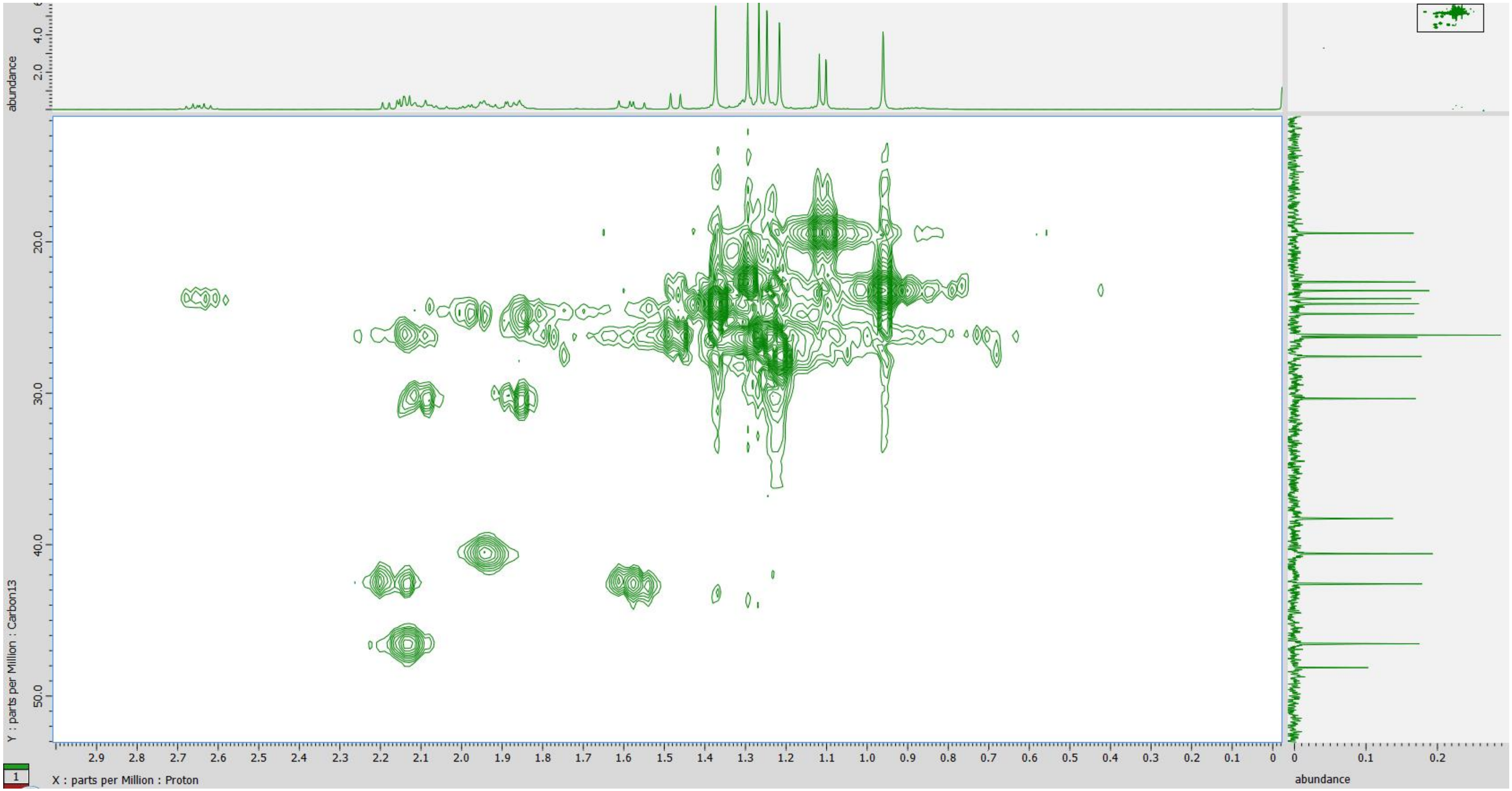

Figure S6

S-4-Me-Ficifolidione (**5**) HMBC

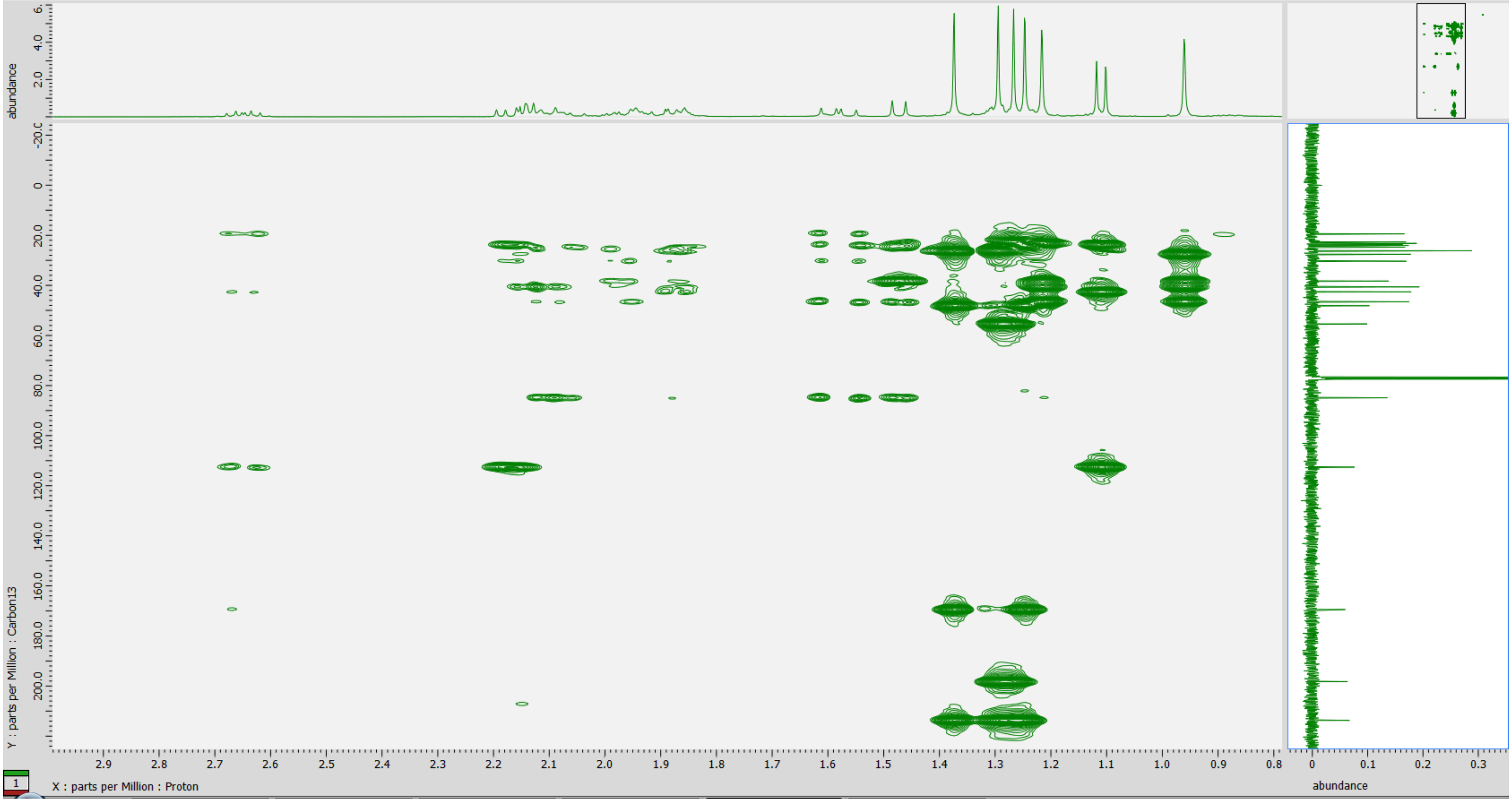

Figure S7

R-4-Me-Ficifolidione (**6**) H-NMR

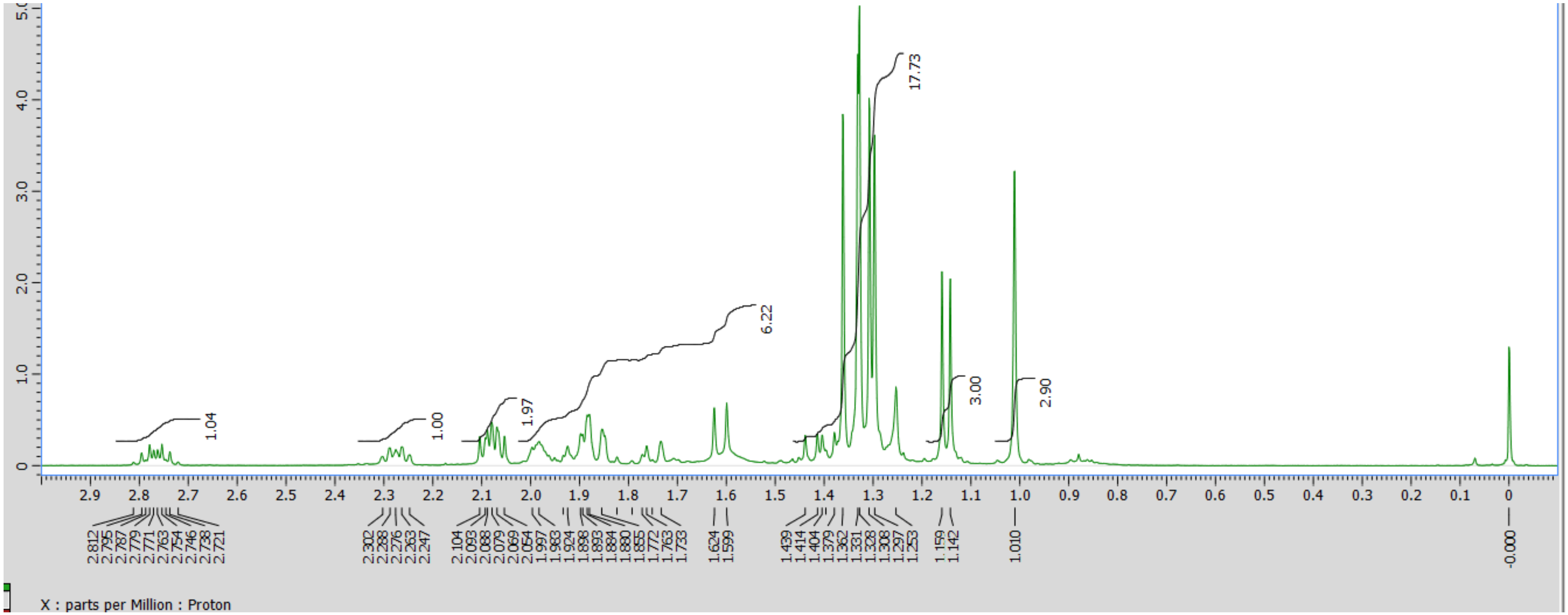

Figure S8

R-4-Me-Ficifolidione (**6**) C-NMR

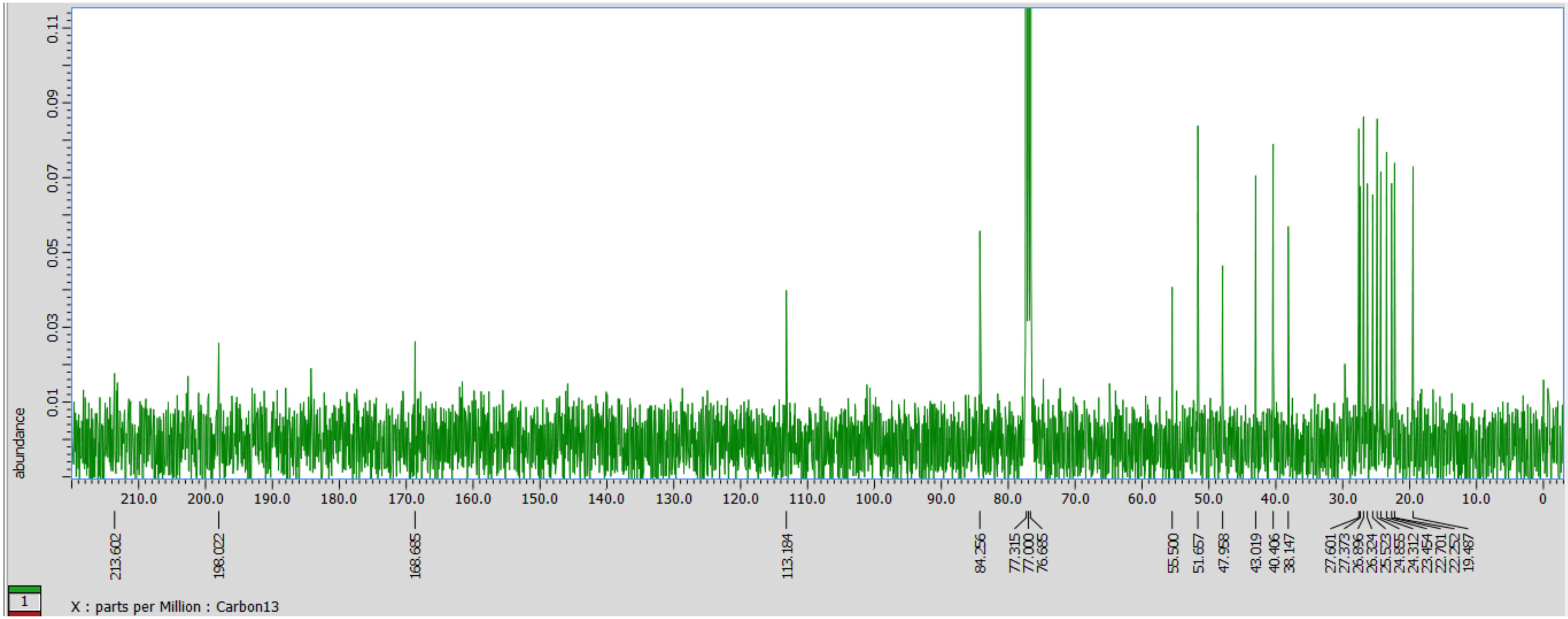

Figure S9

R-4-Me-Ficifolidione (**6**) DEPT

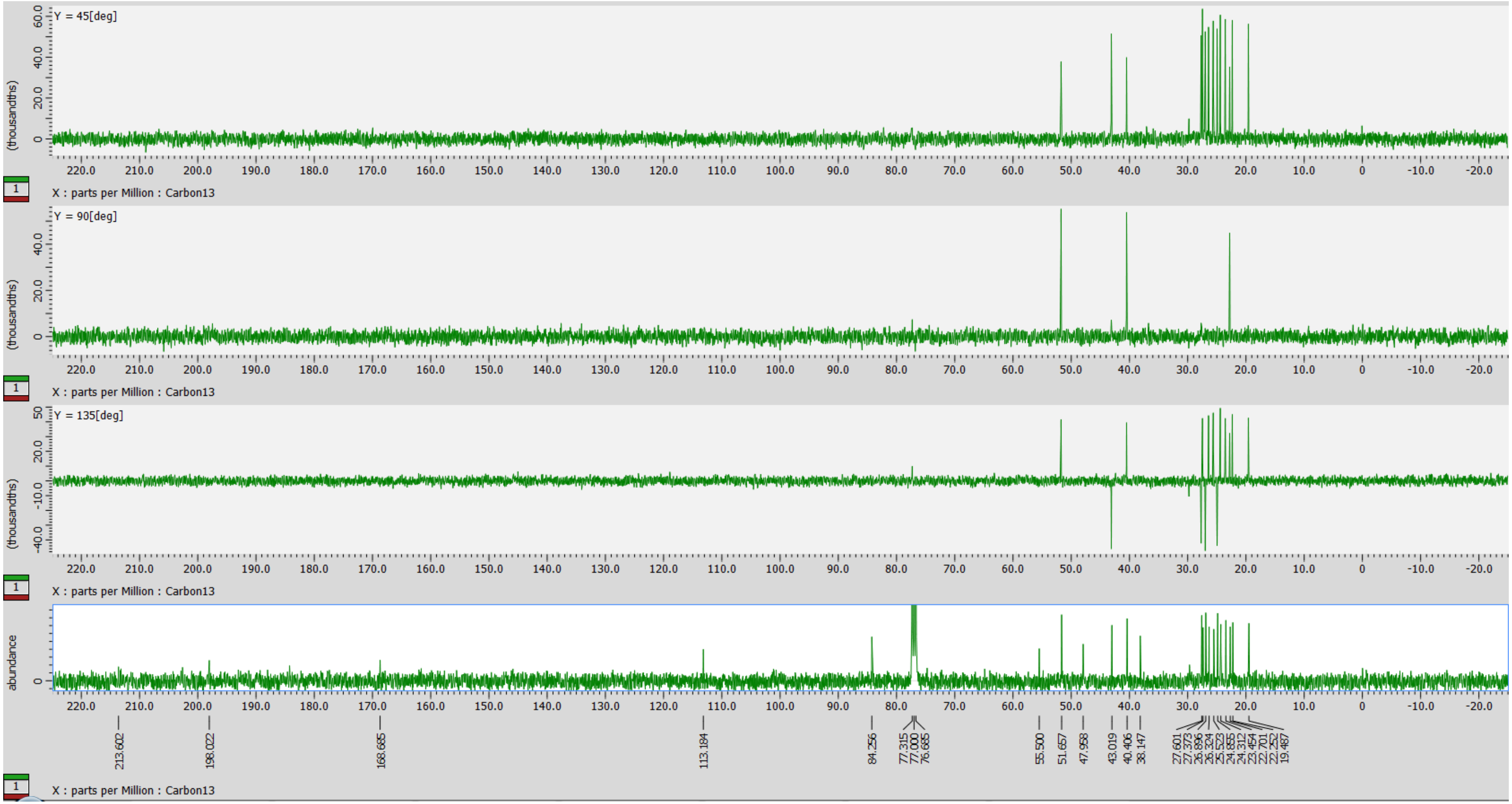

Figure S10

R-4-Me-Ficifolidione (**6**) DEPT expanded

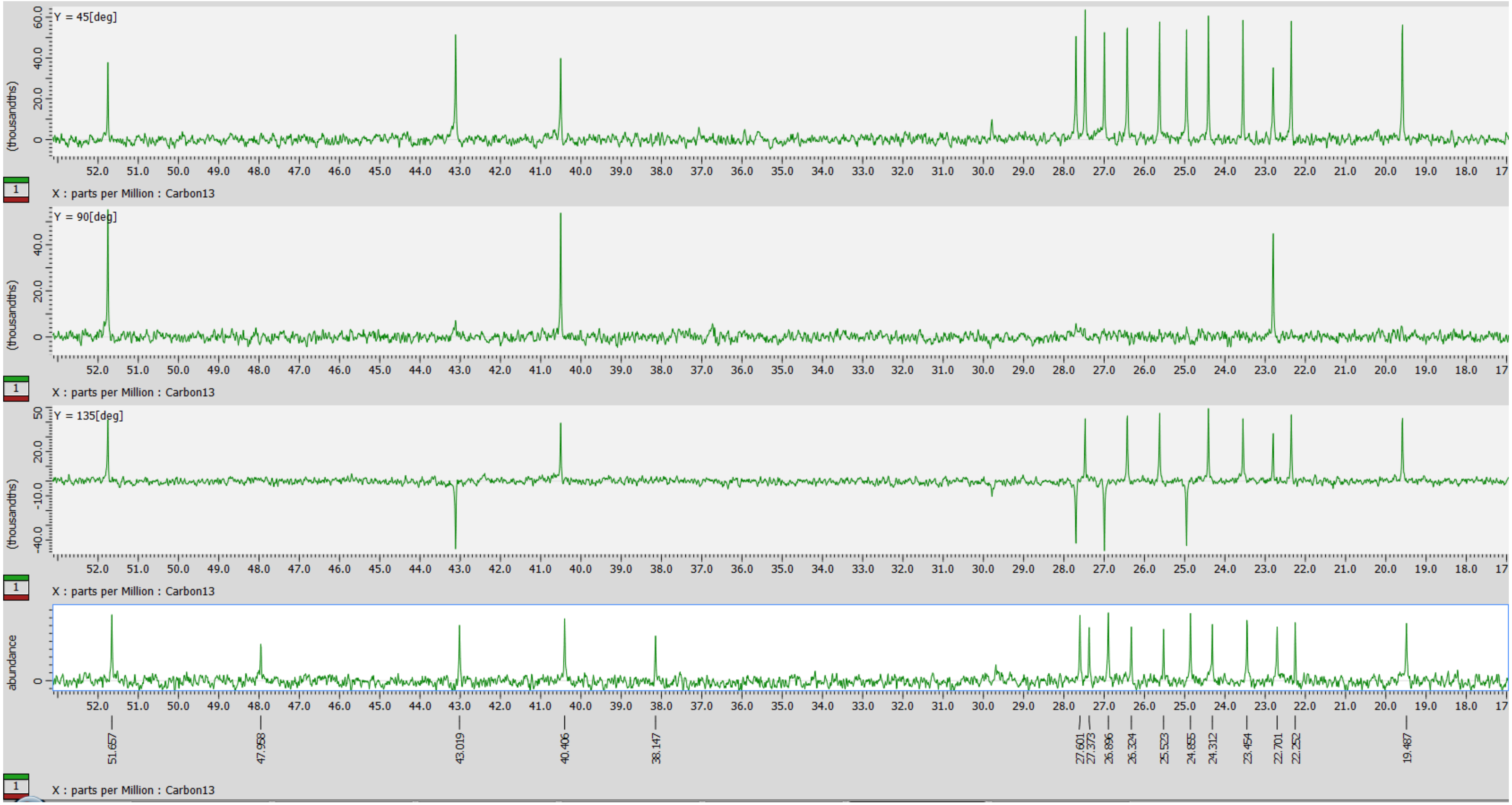

Figure S11

R-4-Me-Ficifolidione (**6**) HMQC

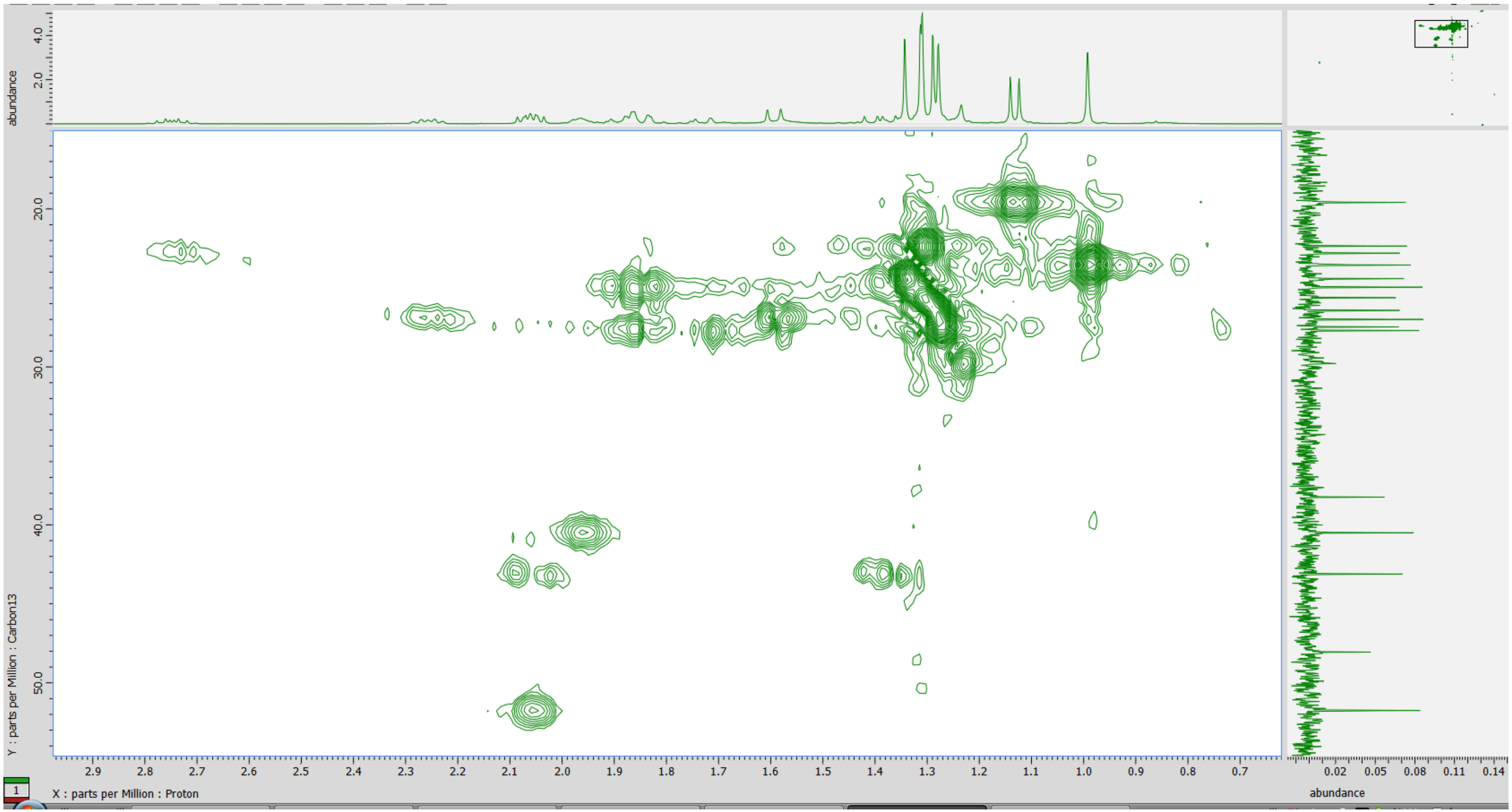

Figure S12

R-4-Me-Ficifolidione (**6**) HMBC

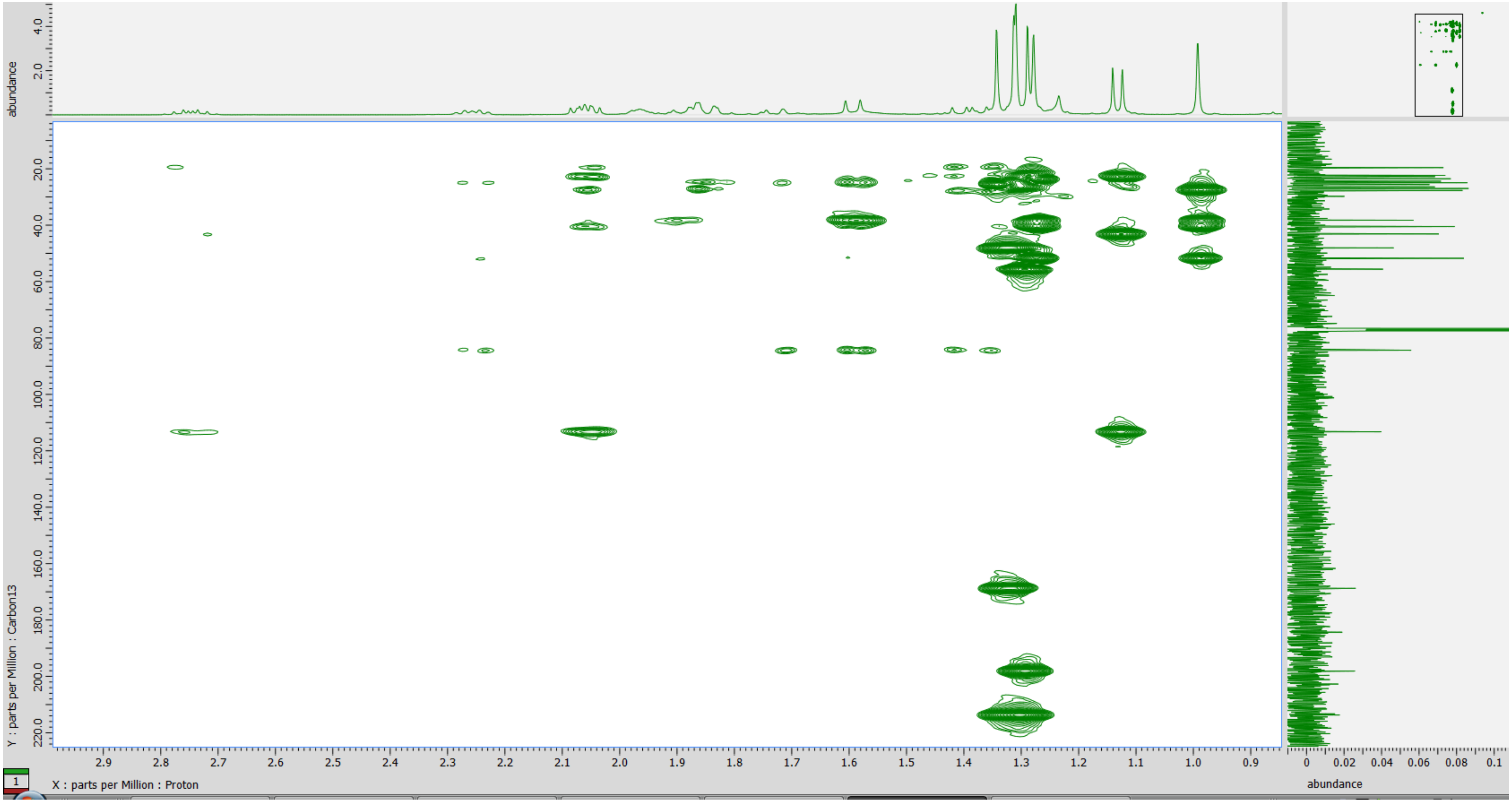

Figure S13

S-4-Pentyl-Ficifolidione (**7**) H-NMR

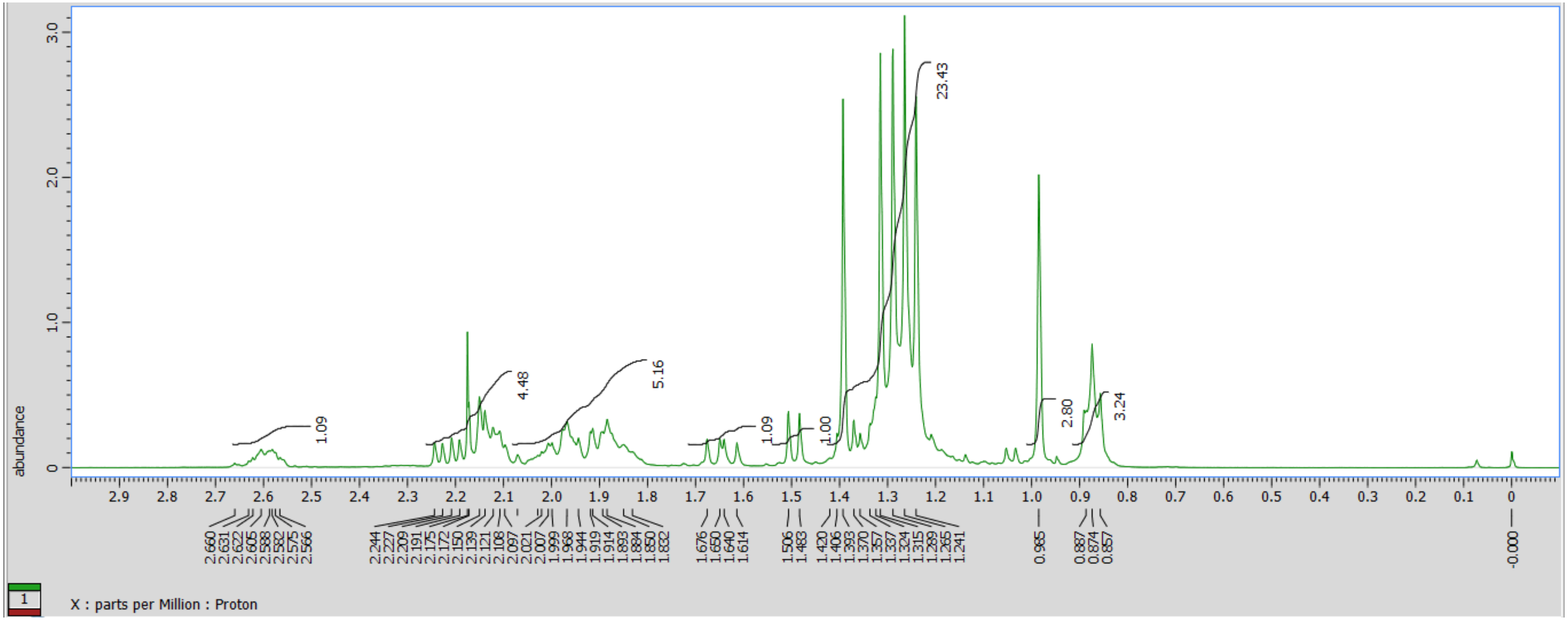

Figure S14

S-4-Pentyl-Ficifolidione (**7**) C-NMR

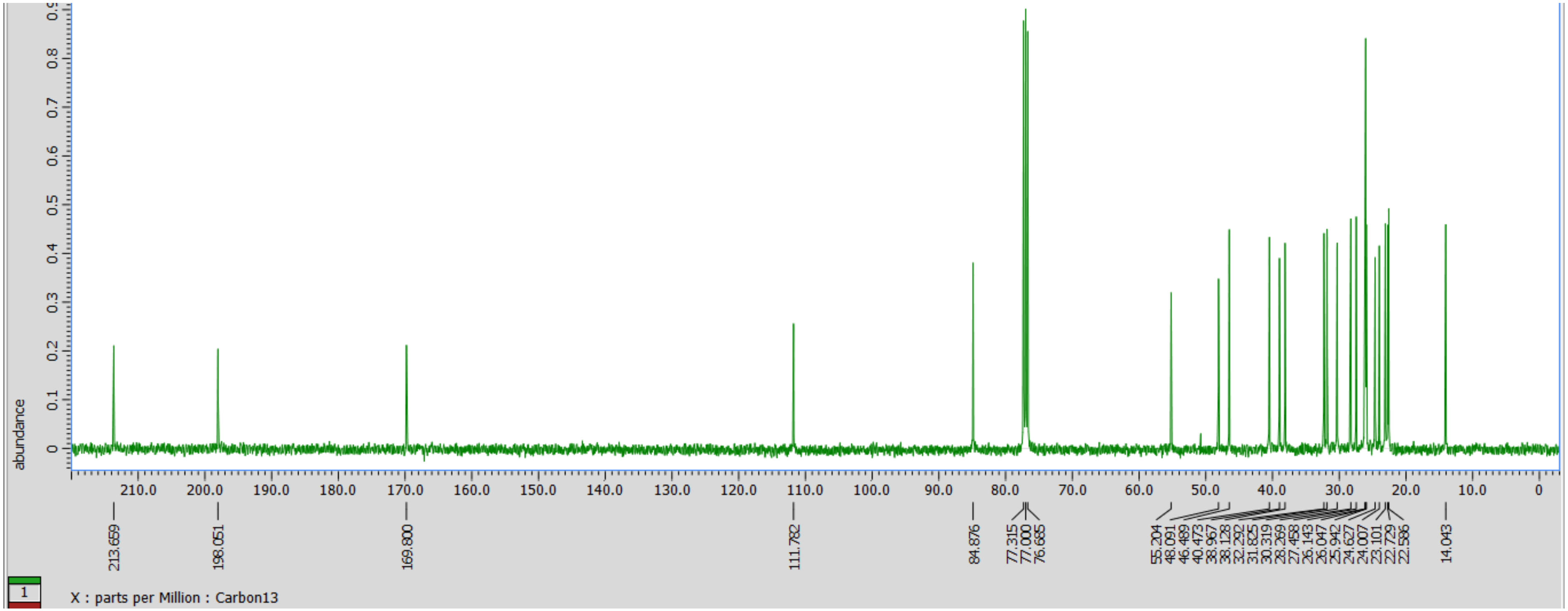

Figure S15

S-4-Pentyl-Ficifolidione (**7**) DEPT

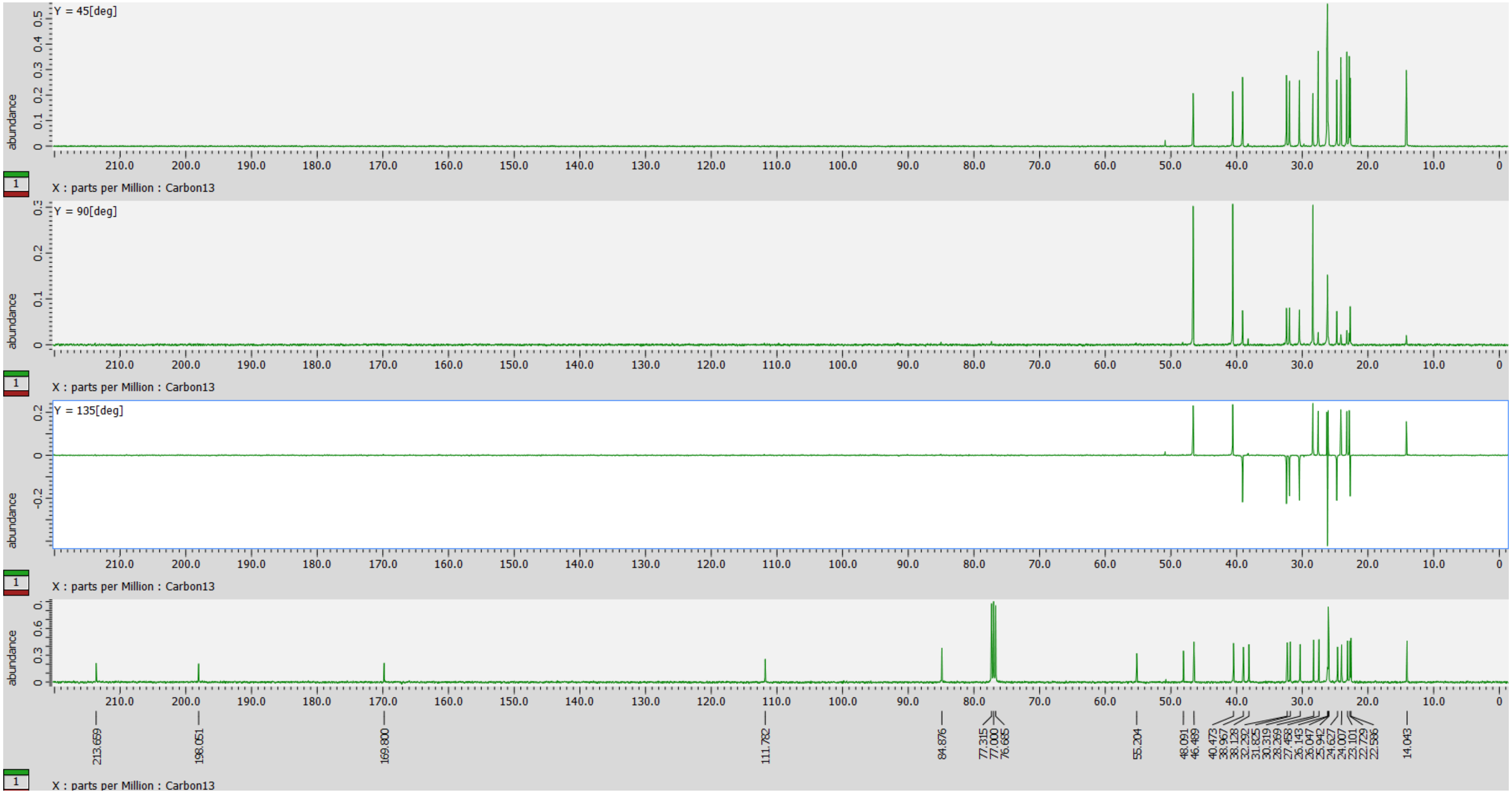

Figure S16

S-4-Pentyl-Ficifolidione (**7**) DEPT expanded

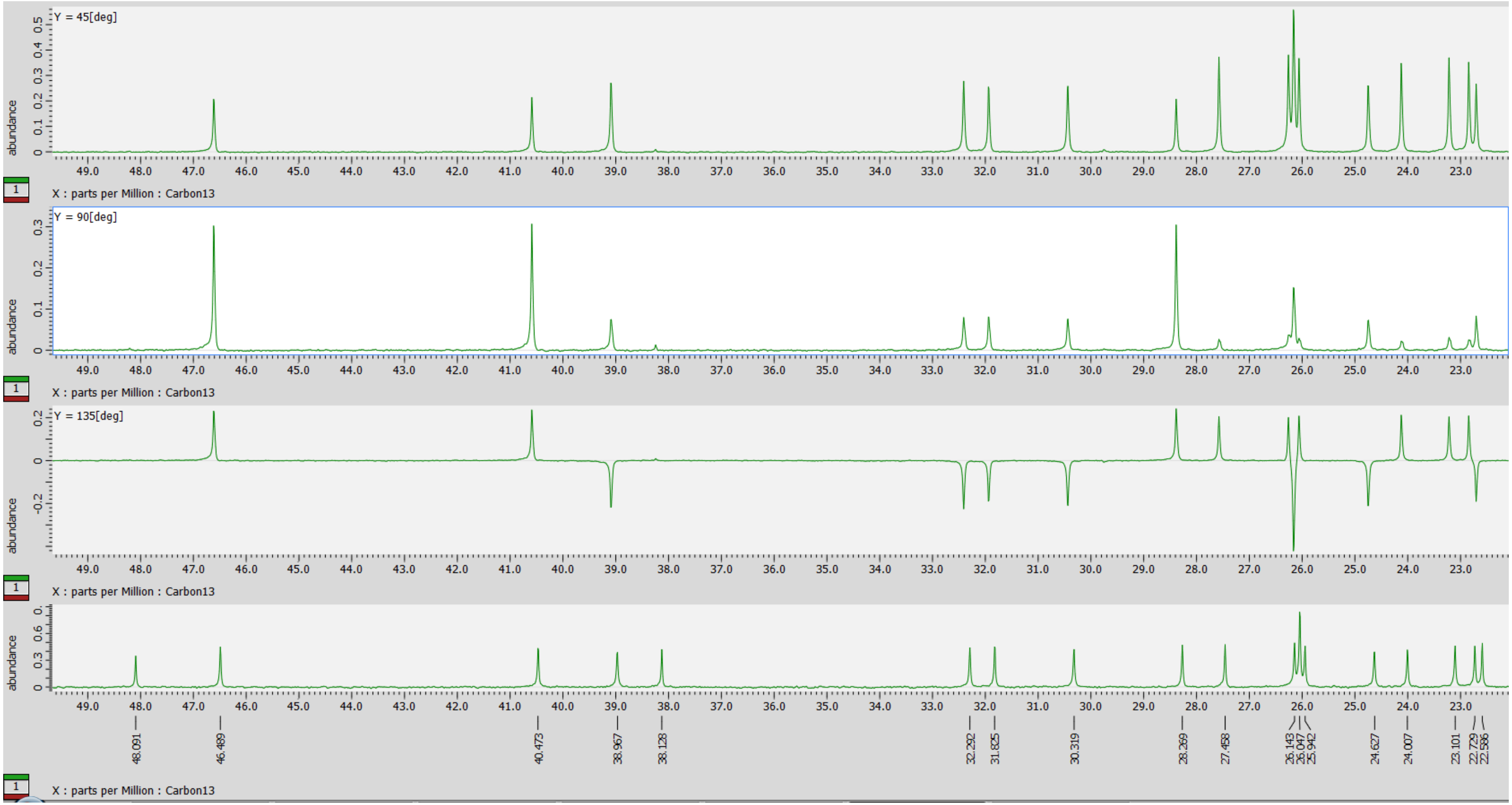

Figure S17

S-4-Pentyl-Ficifolidione (**7**) HMQC

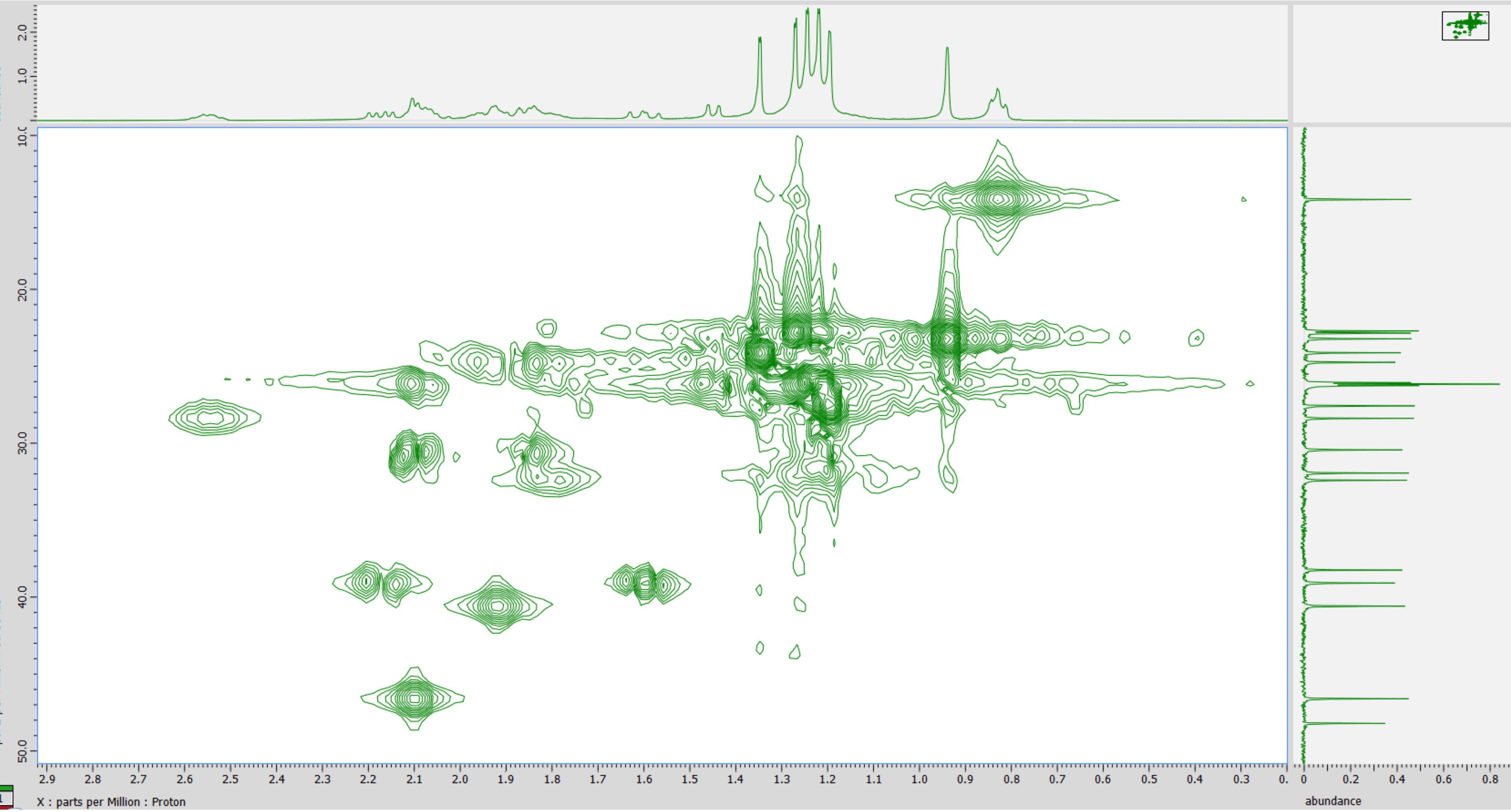

Figure S18

S-4-Pentyl-Ficifolidione (**7**) HMBC

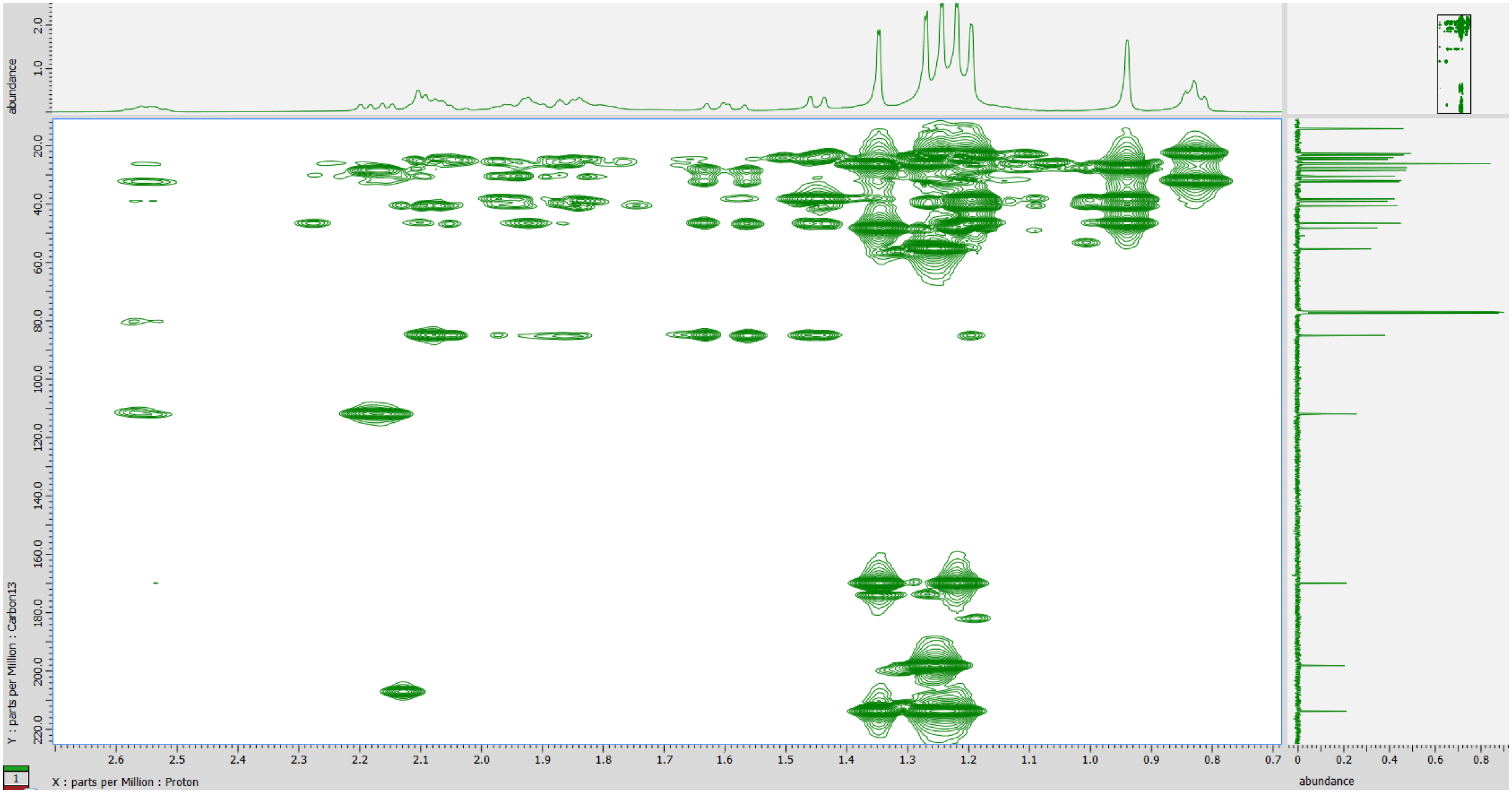

Figure S19

R-4-Pentyl-Ficifolidione (**8**) H-NMR

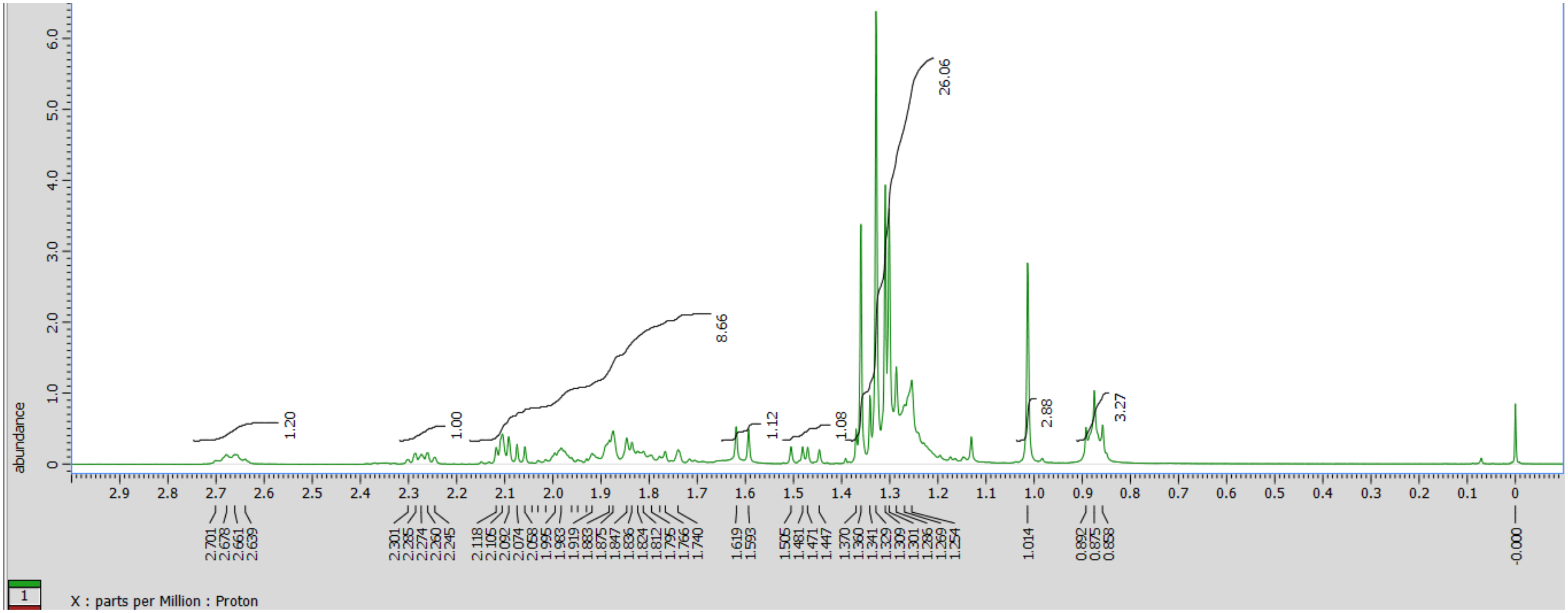

Figure S20

R-4-Pentyl-Ficifolidione (**8**) C-NMR

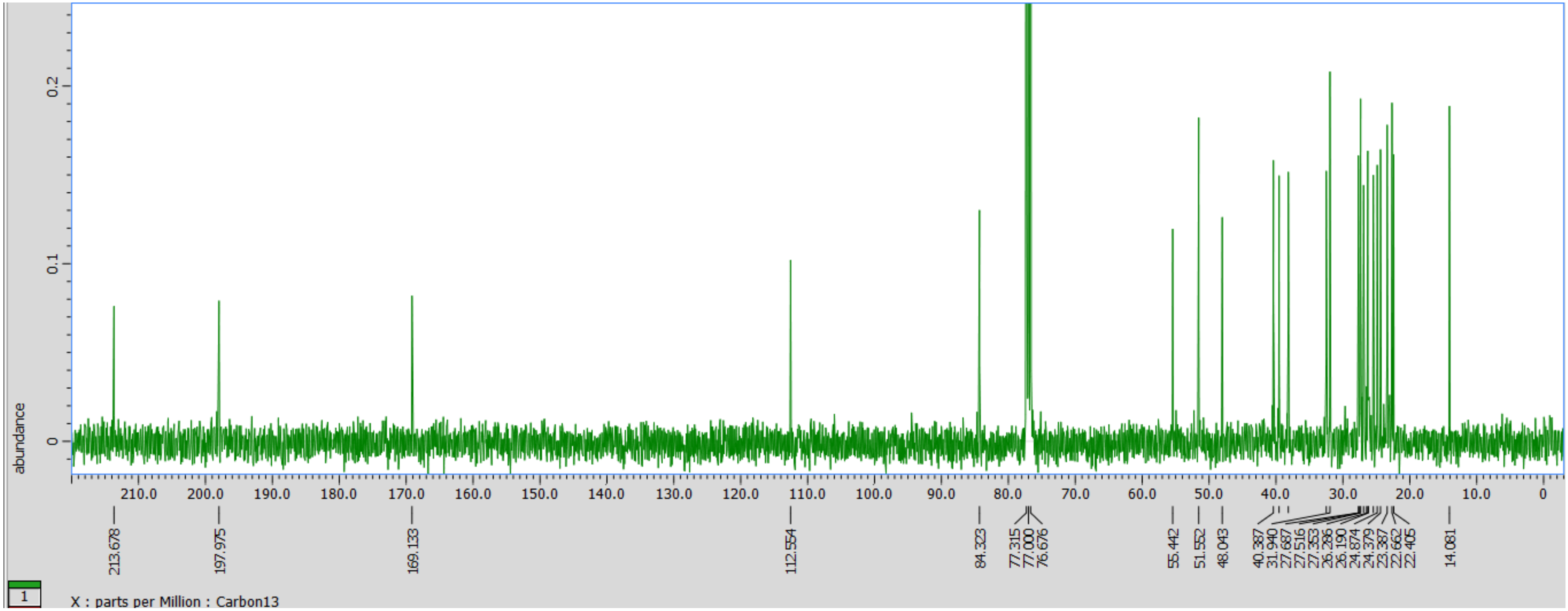

Figure S21

R-4-Pentyl-Ficifolidione (**8**) DEPT

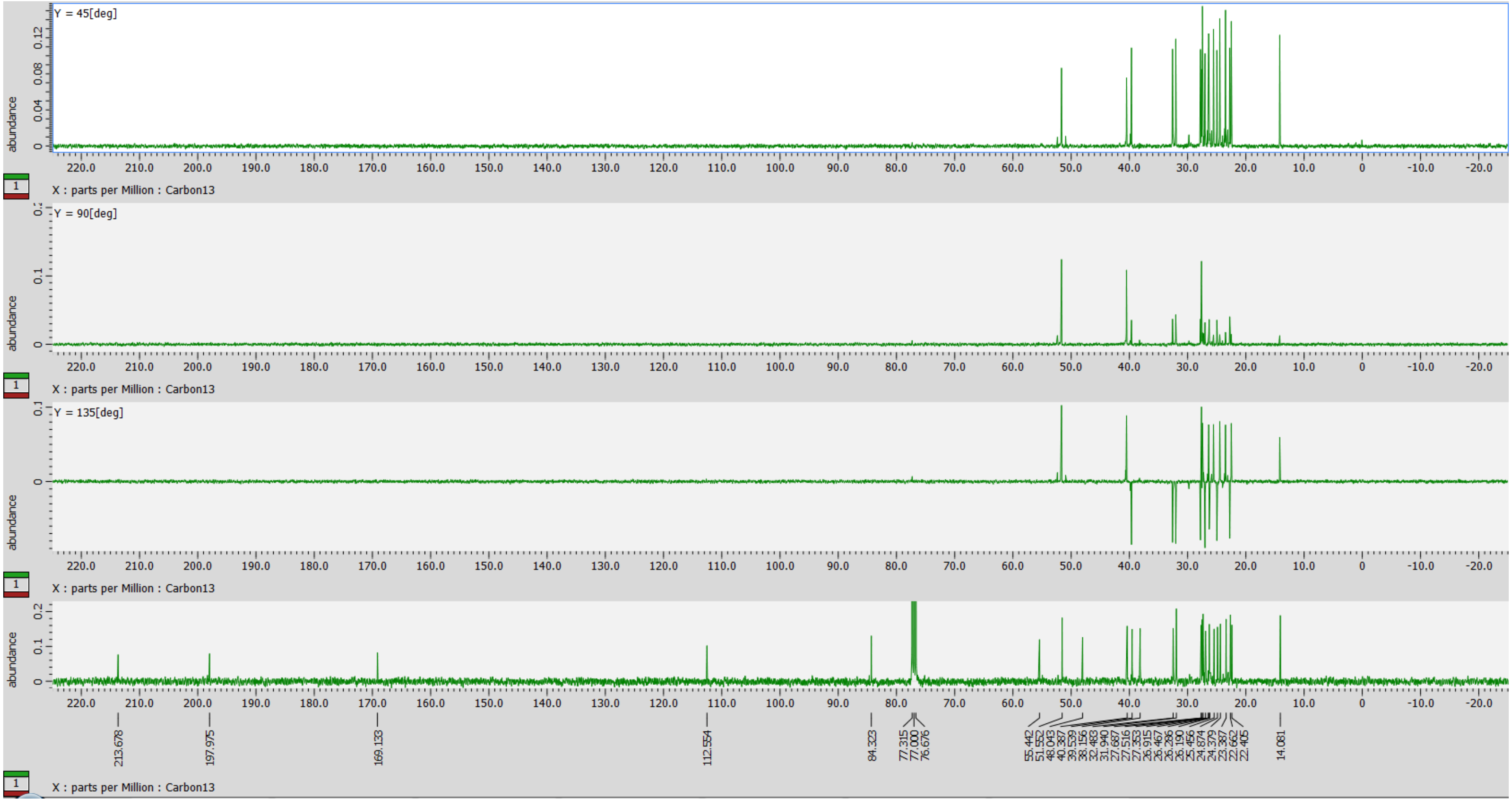

Figure S22

R-4-Pentyl-Ficifolidione (**8**) DEPT expanded

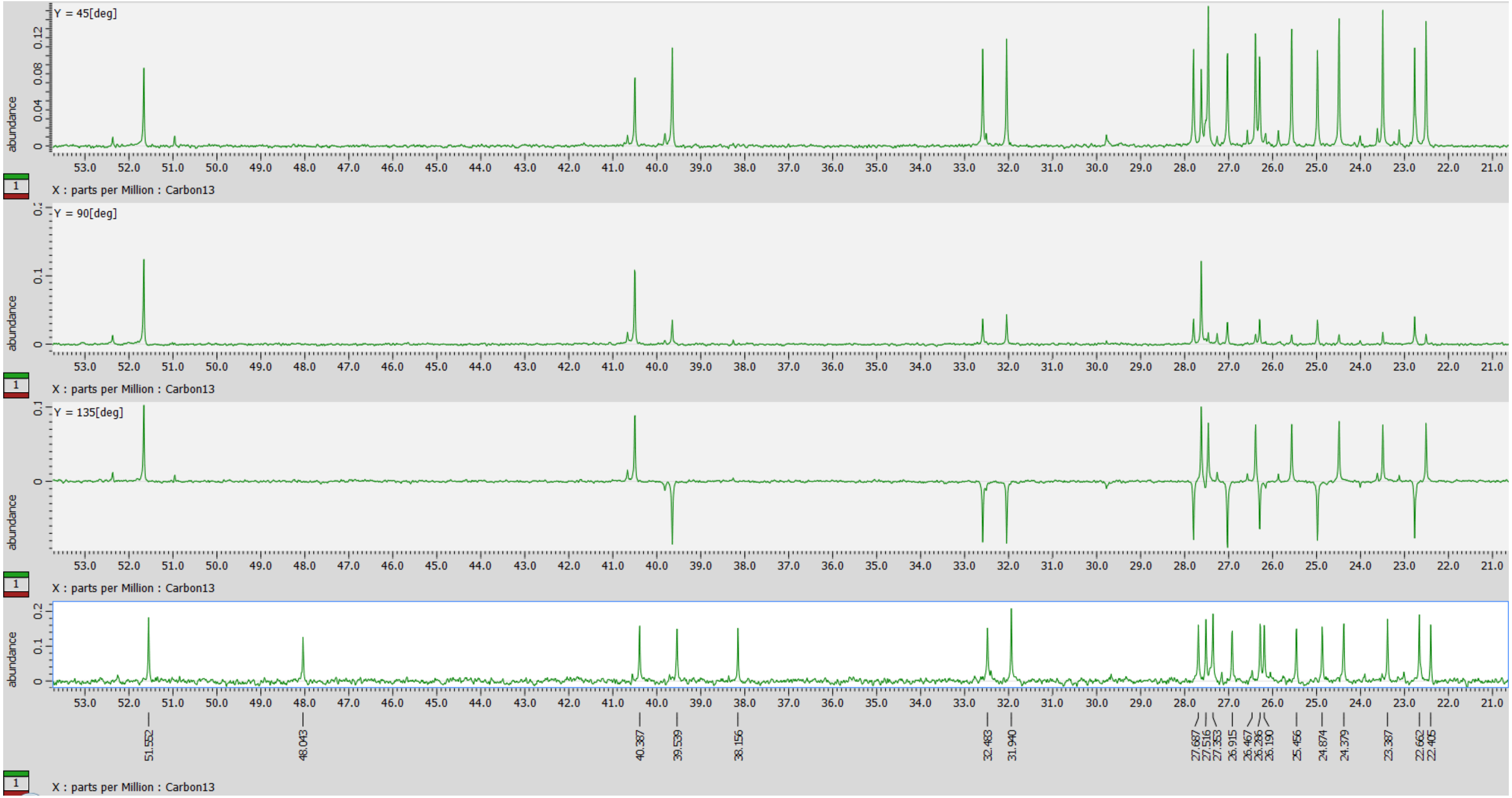

Figure S23

R-4-Pentyl-Ficifolidione (**8**) HMQC

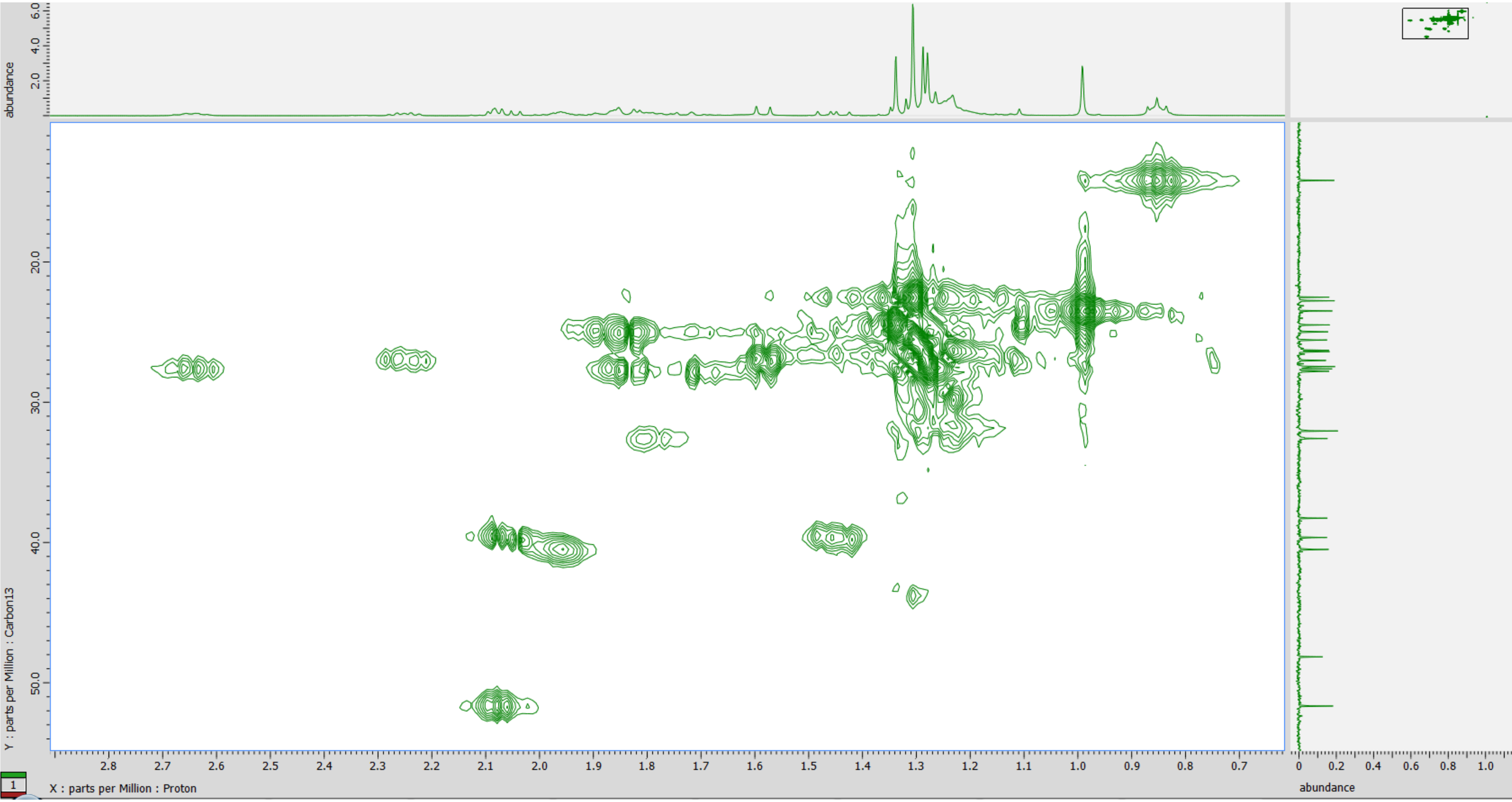

Figure S24

R-4-Pentyl-Ficifolidione (**8**) HMBC

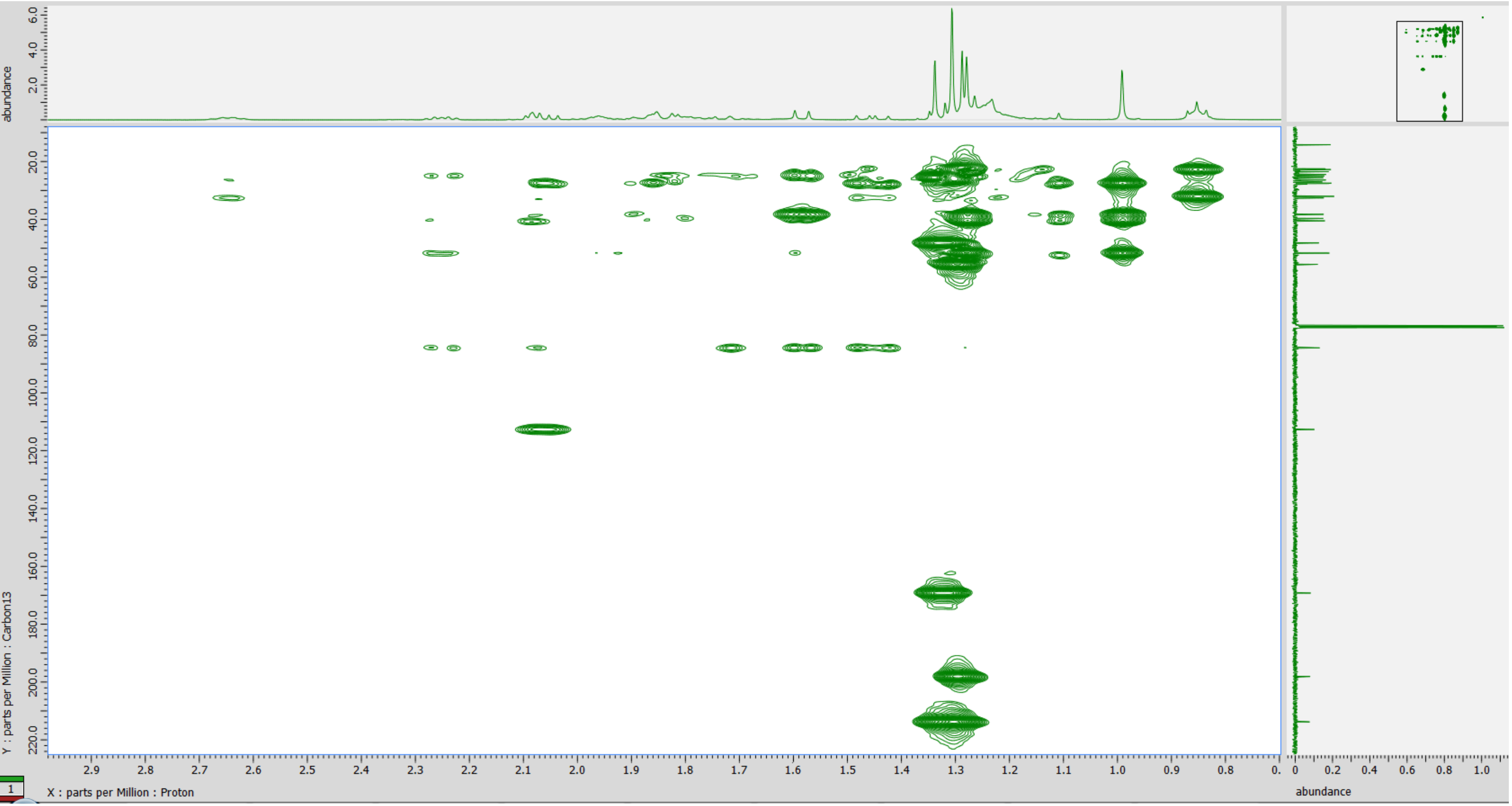

Figure S25

S-4-Heptyl-Ficifolidione (**9**) H-NMR

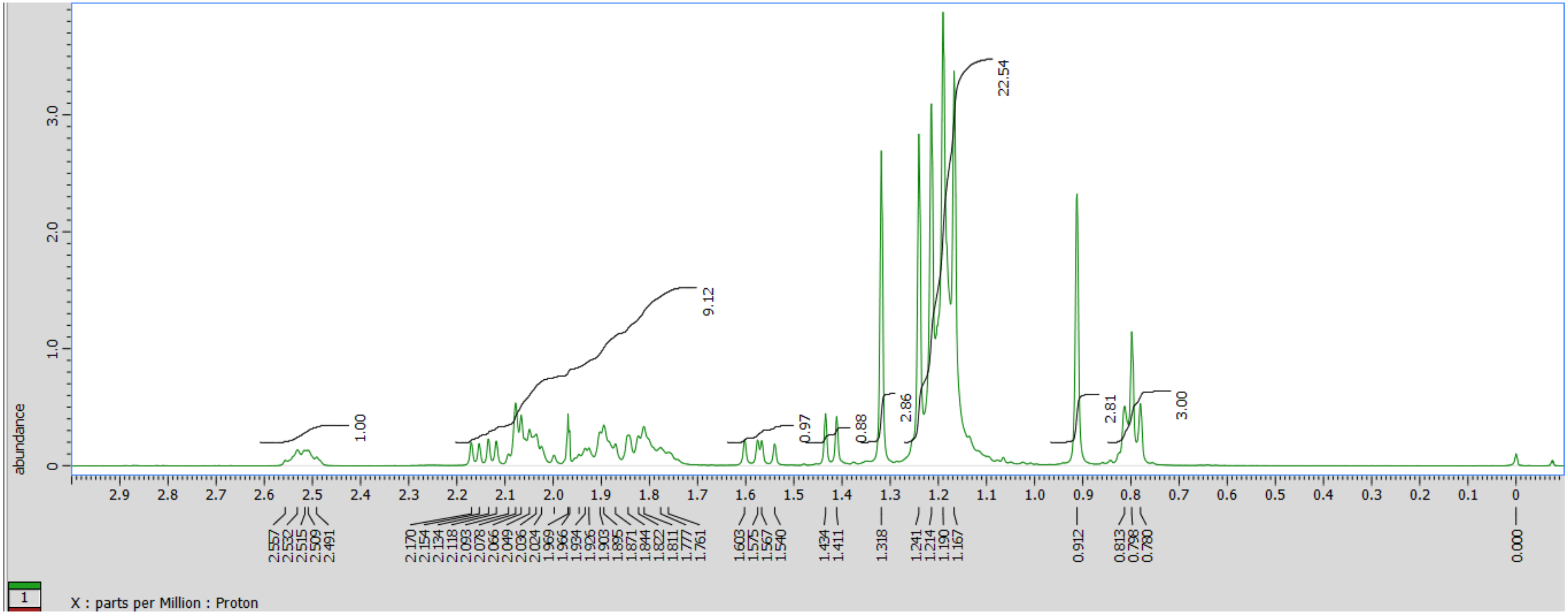

Figure S26

S-4-Heptyl-Ficifolidione (**9**) C-NMR

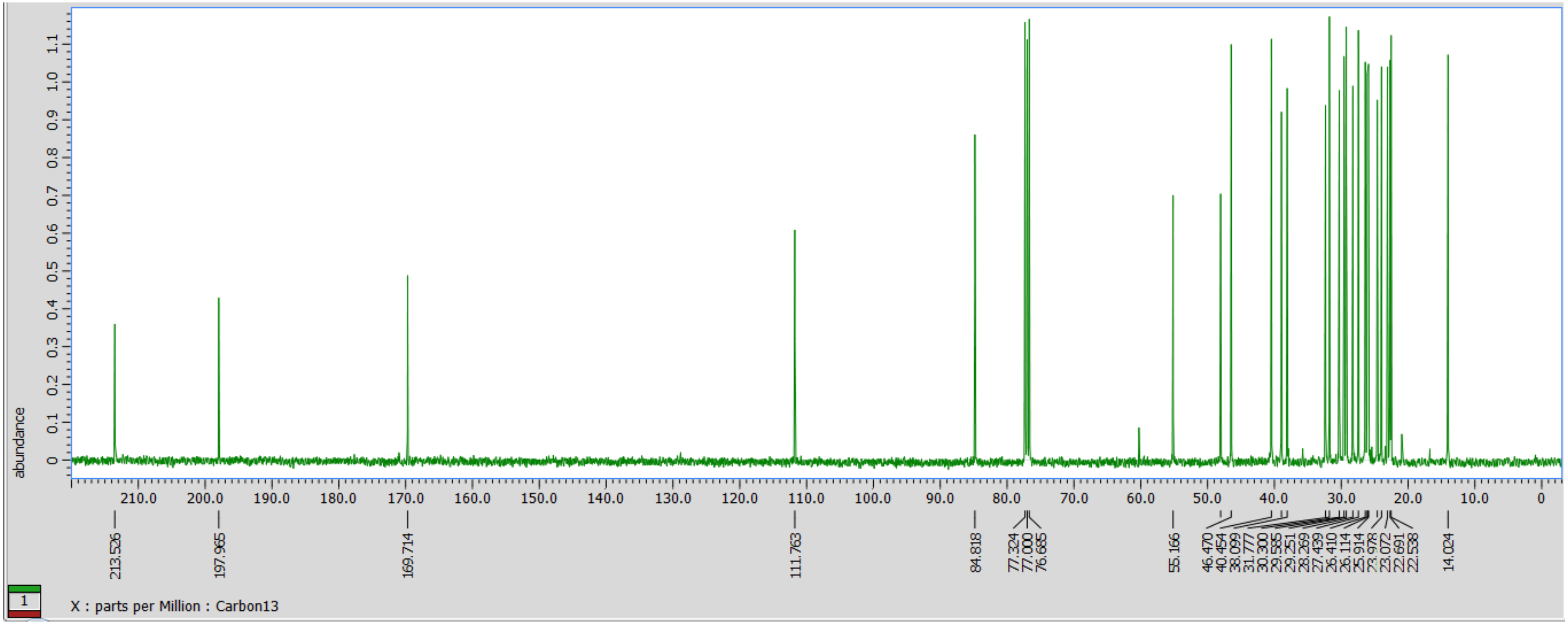

Figure S27

S-4-Heptyl-Ficifolidione (**9**) DEPT

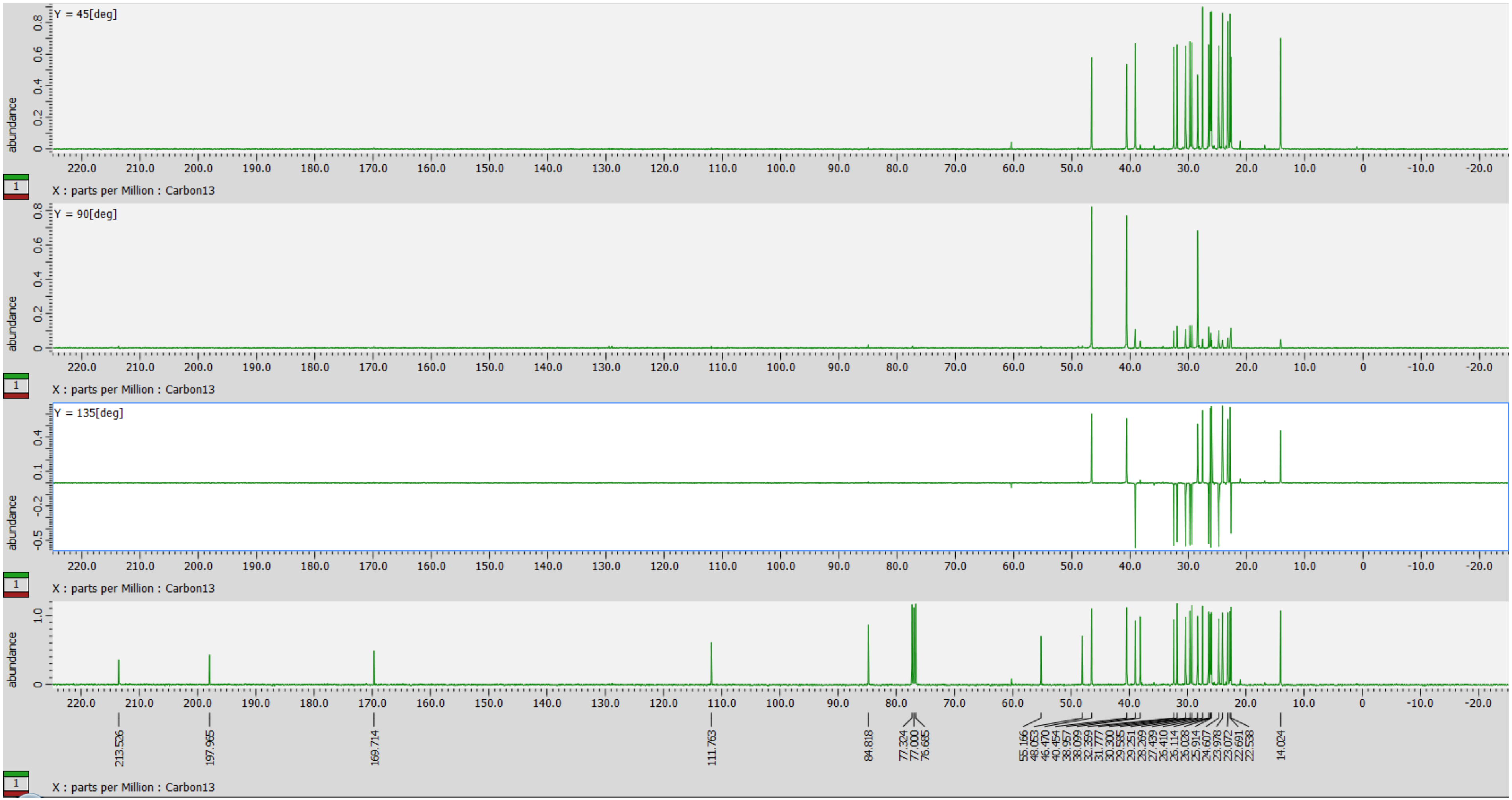

Figure S28

S-4-Heptyl-Ficifolidione (**9**) DEPT expanded

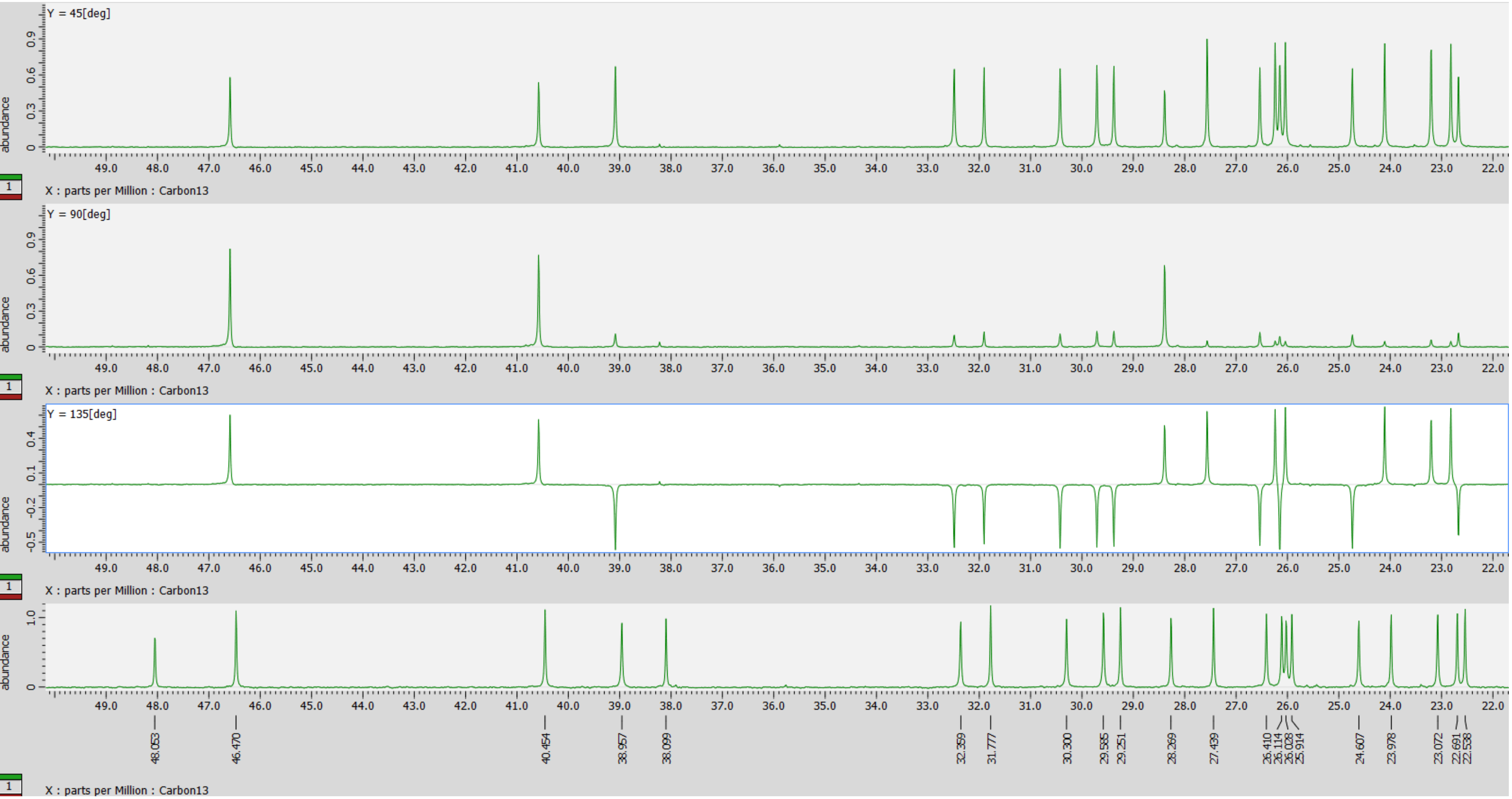

Figure S29

S-4-Heptyl-Ficifolidione (**9**) HMQC

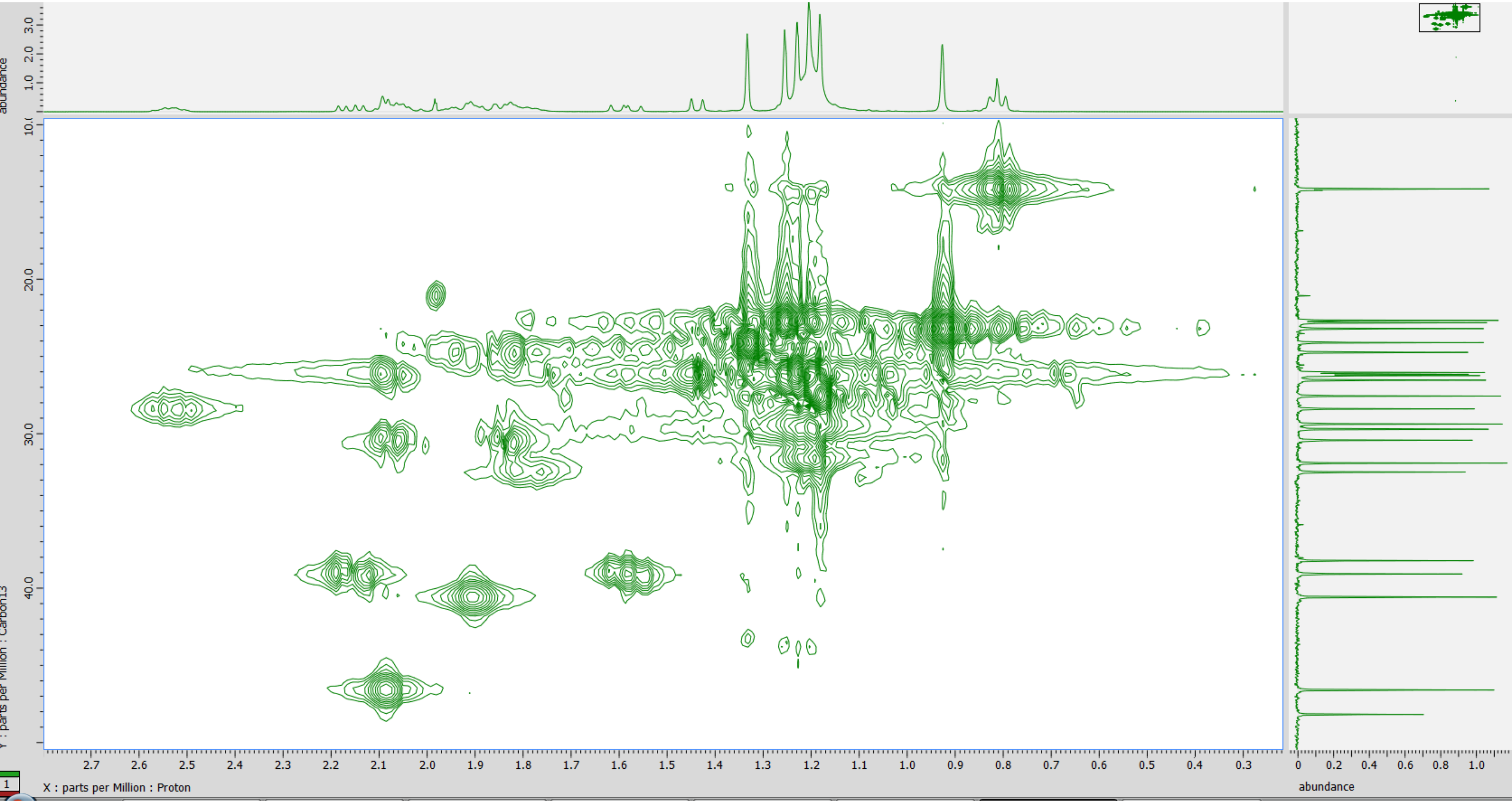

Figure S30

S-4-Heptyl-Ficifolidione (**9**) HMBC

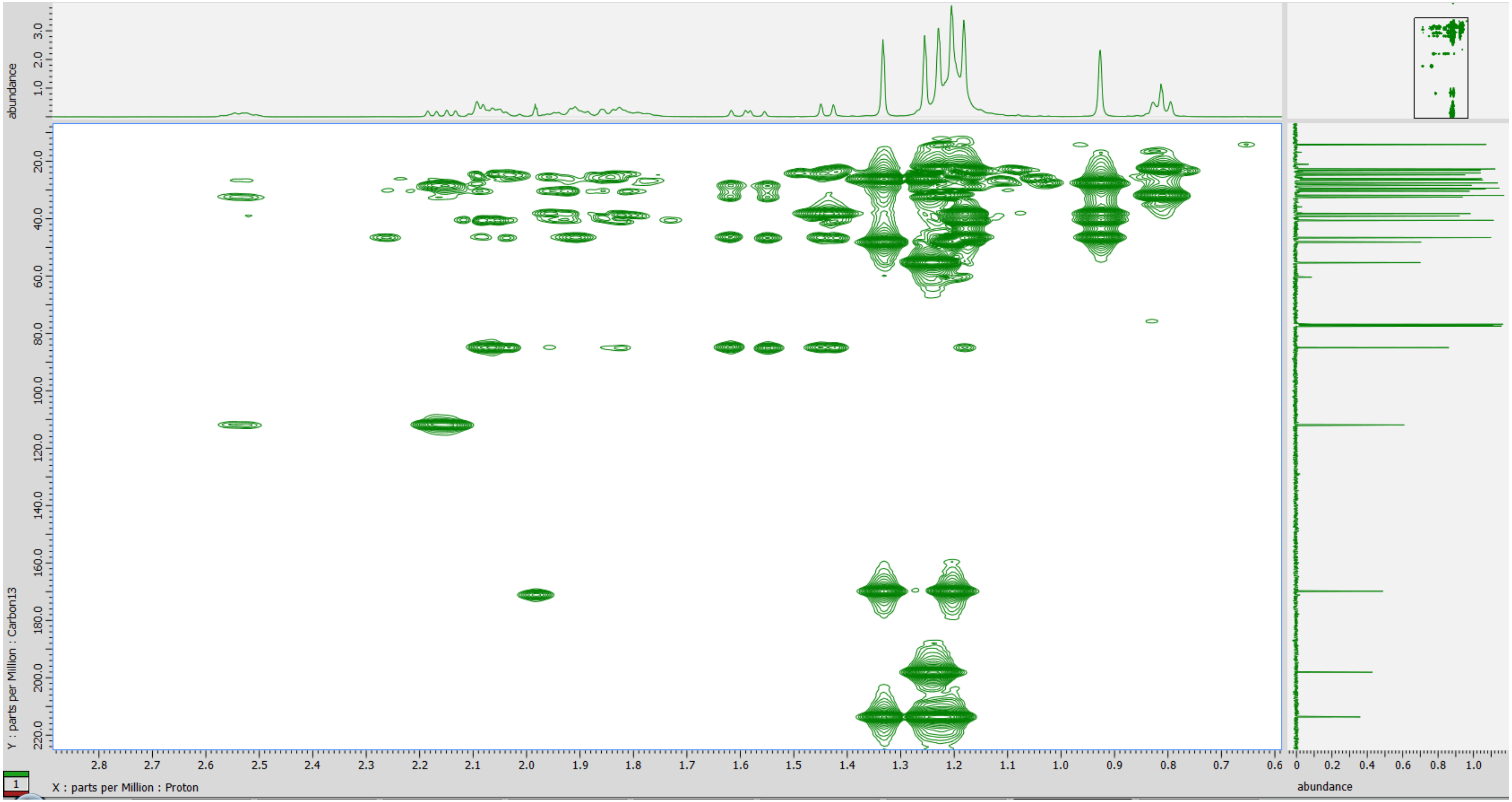

Figure S31

R-4-Heptyl-Ficifolidione (**10**) H-NMR

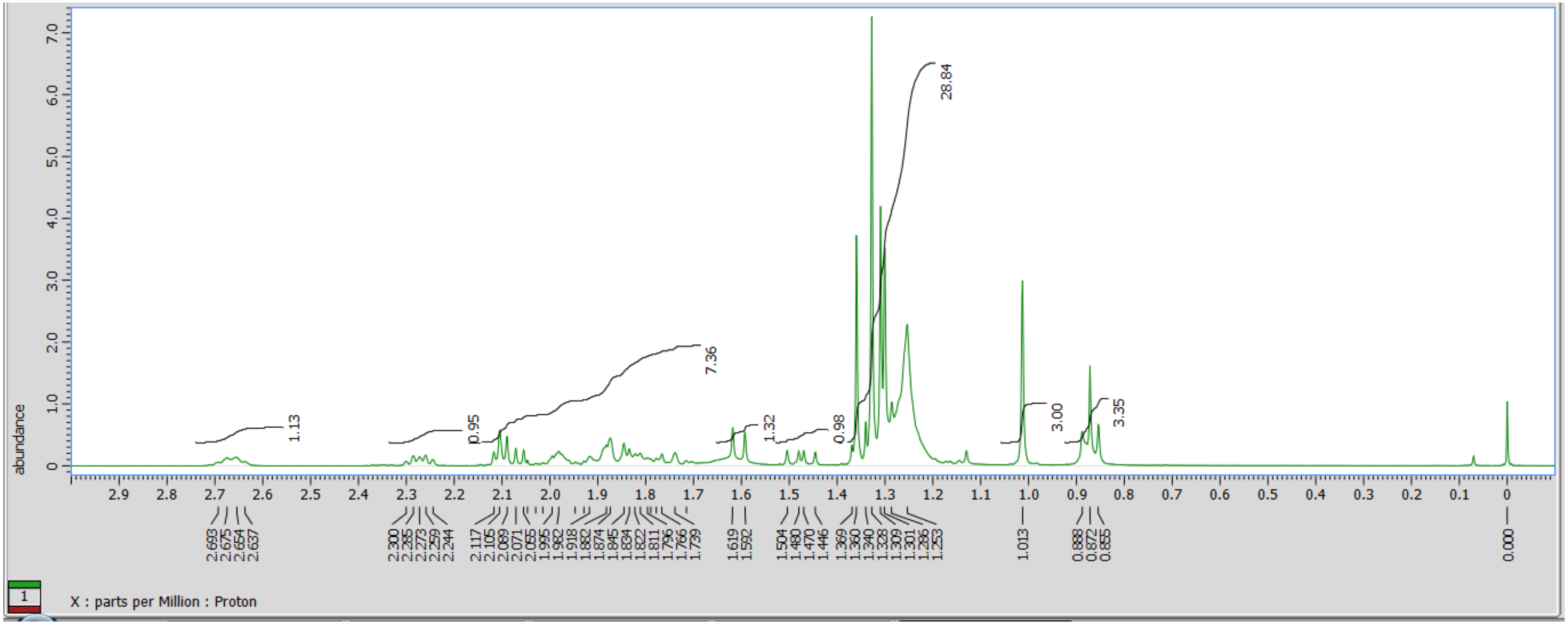

Figure S32

R-4-Heptyl-Ficifolidione (**10**) C-NMR

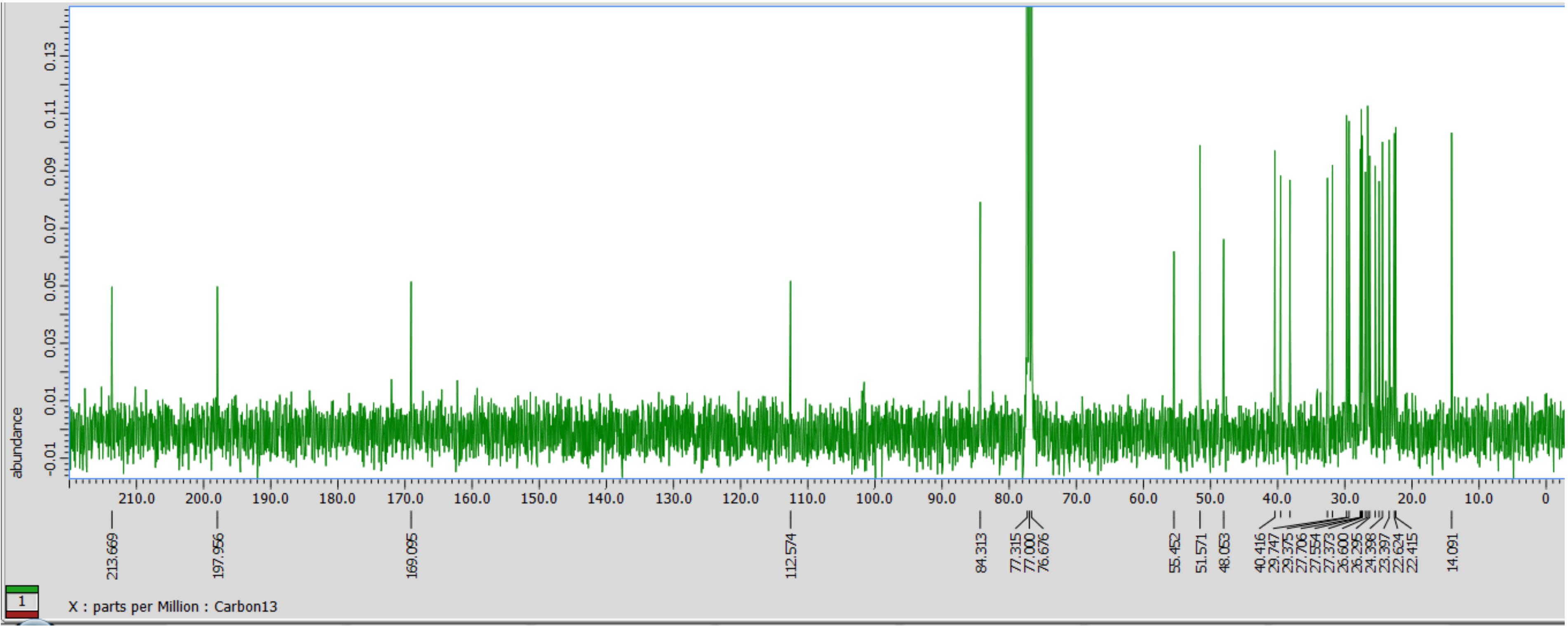

Figure S33

R-4-Heptyl-Ficifolidione (**10**) DEPT

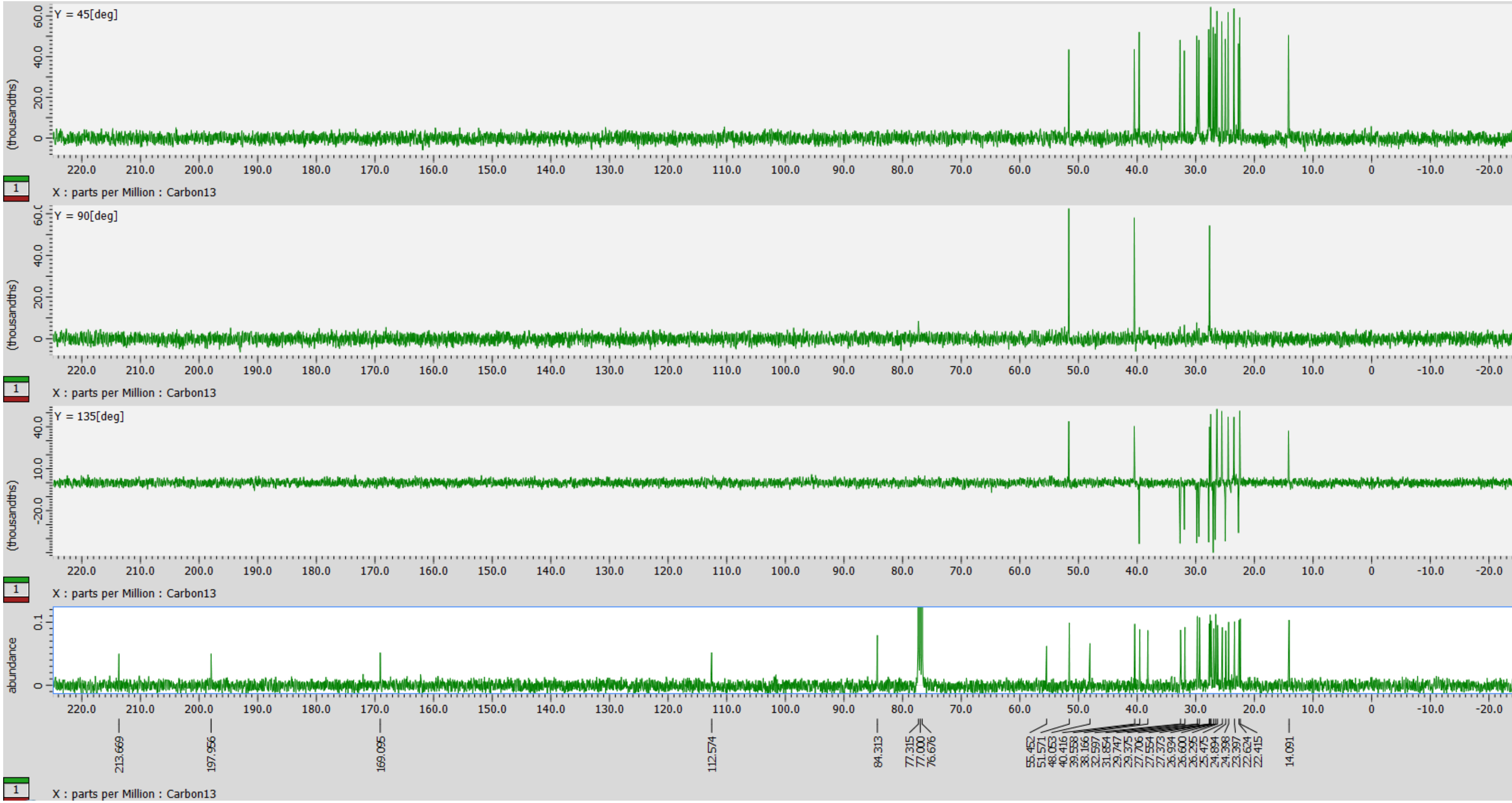

Figure S34

R-4-Heptyl-Ficifolidione (**10**) DEPT expanded

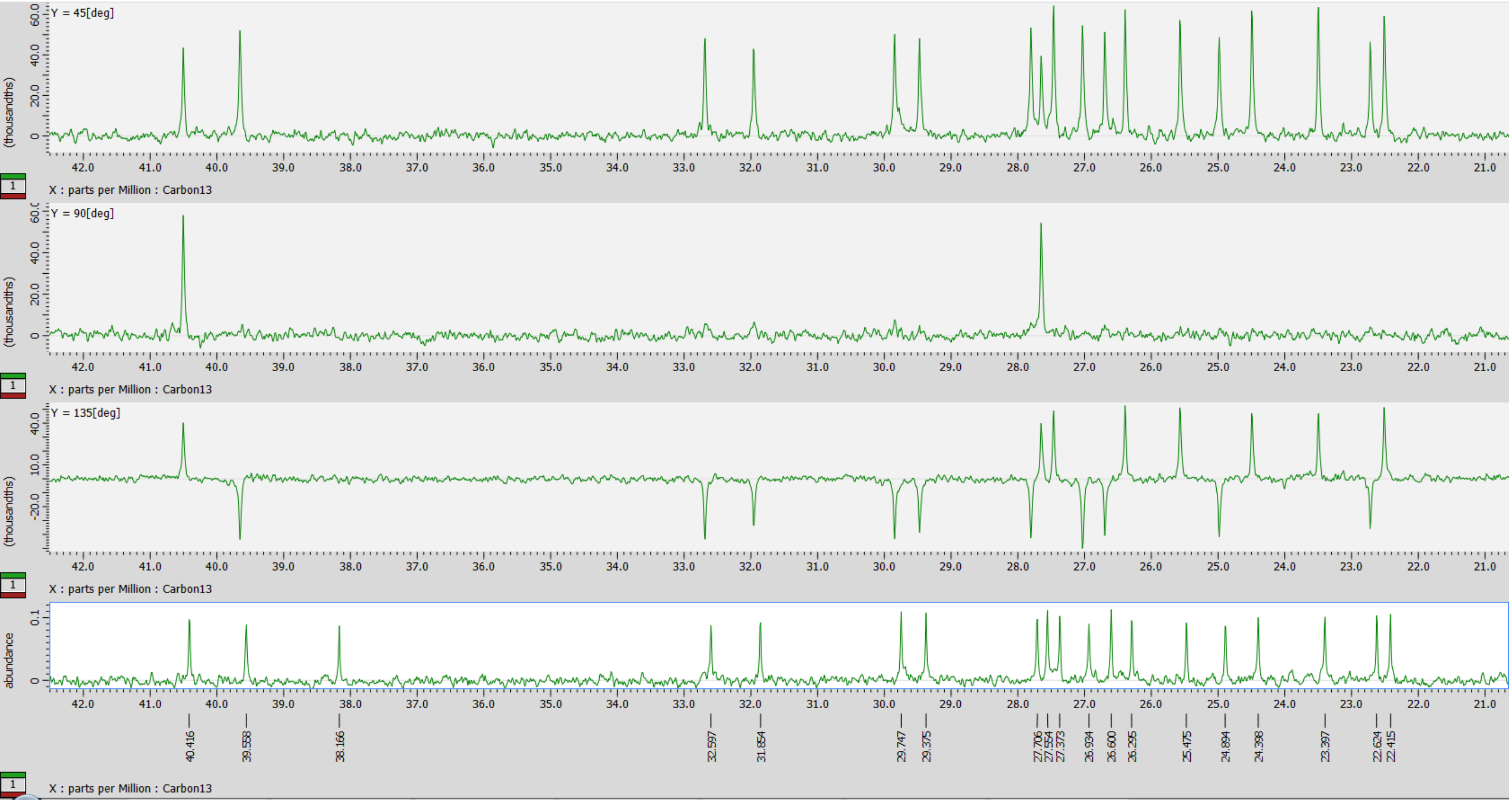

Figure S35

R-4-Heptyl-Ficifolidione (**10**) HMQC

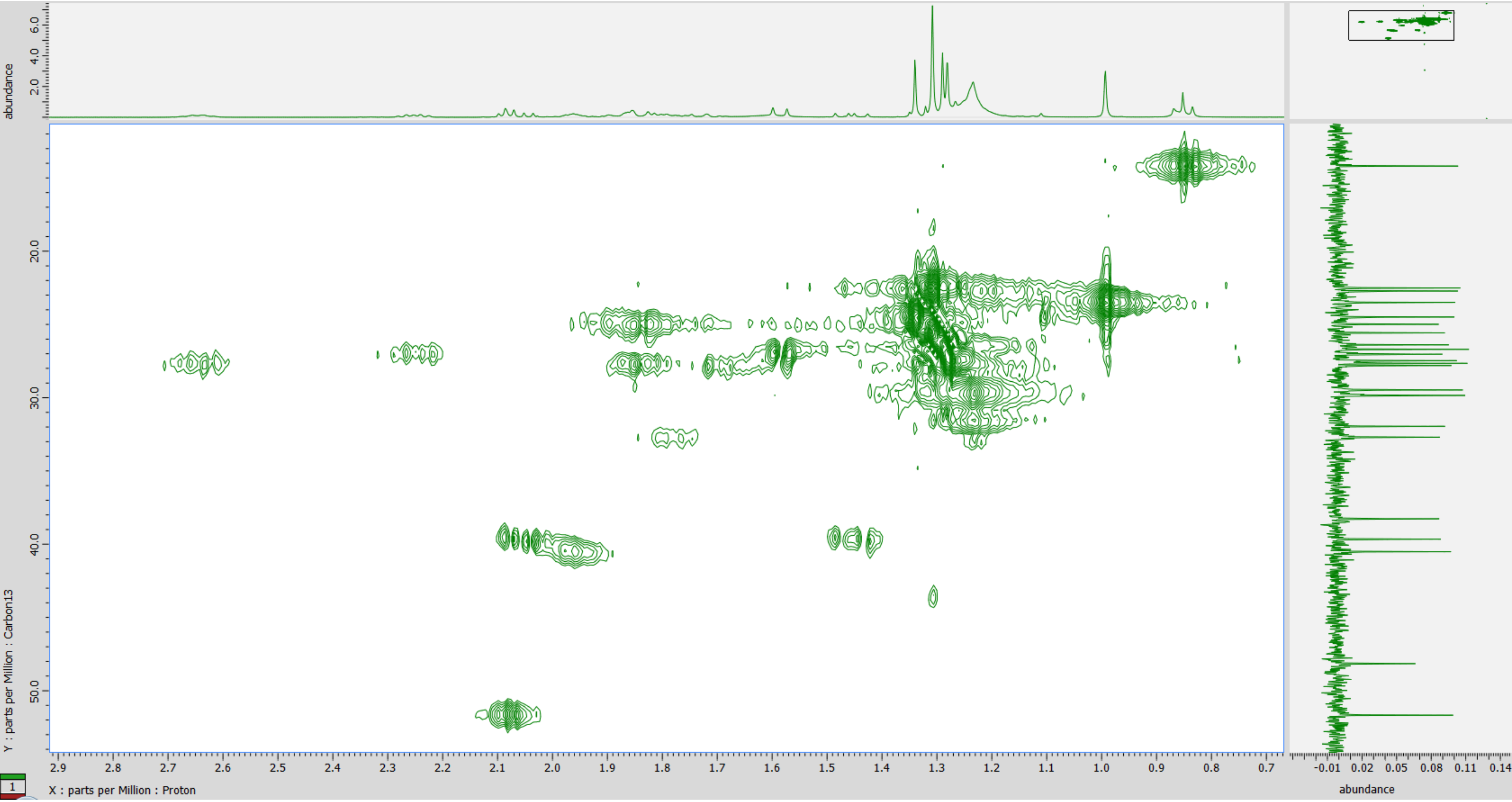

Figure S36

R-4-Heptyl-Ficifolidione (**10**) HMBC

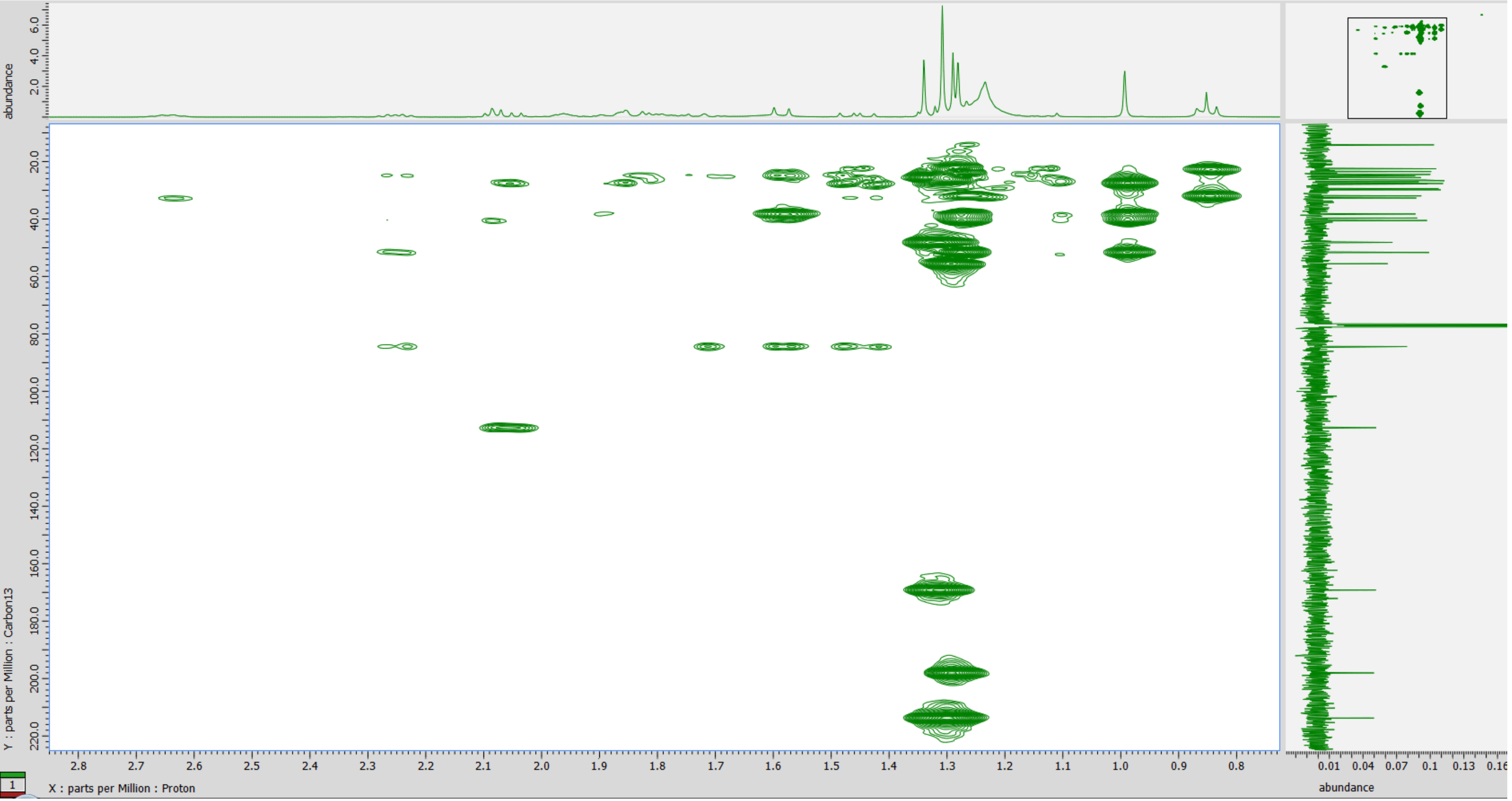

Supplement: Supplementary file 1 [file molecules-24-04081-s001.pdf]
